# Low-dose acetaminophen induces early disruption of cell-cell tight junctions in human hepatic cells and mouse liver

**DOI:** 10.1038/srep37541

**Published:** 2017-01-30

**Authors:** Wesam Gamal, Philipp Treskes, Kay Samuel, Gareth J. Sullivan, Richard Siller, Vlastimil Srsen, Katie Morgan, Anna Bryans, Ada Kozlowska, Andreas Koulovasilopoulos, Ian Underwood, Stewart Smith, Jorge del-Pozo, Sharon Moss, Alexandra Inés Thompson, Neil C. Henderson, Peter C. Hayes, John N. Plevris, Pierre-Olivier Bagnaninchi, Leonard J. Nelson

**Affiliations:** 1 MRC Centre for Regenerative Medicine, SCRM Building, The University of Edinburgh, Edinburgh BioQuarter, 5 Little France Drive, Edinburgh EH16 4UU, UK; 2 Hepatology Laboratory, University of Edinburgh, Royal Infirmary of Edinburgh, 49 Little France Crescent EH16 4SB, UK; 3 Scottish National Blood Transfusion Service, Research, Development and Innovation Directorate, Cell Therapy Group, Ellens Glen Road, Edinburgh, EH17 7QT, UK; 4 Department of Biochemistry, Institute of Basic Medical Sciences, Faculty of Medicine, University of Oslo, PO Box 1112 Blindern, 0317 Oslo, Norway, UK; 5 Norwegian Center for Stem Cell Research, PO Box 1112 Blindern, 0317 Oslo, Norway; 6 Institute of Immunology, Oslo University Hospital-Rikshospitalet, PO Box 4950 Nydalen, Oslo 0424, Norway; 7 Institute for Bioengineering, University of Edinburgh, King’s Buildings, Colin MacLaurin Road, EH9 3DW, UK; 8 Institute for Integrated Micro and Nano systems, University of Edinburgh, Scottish Micro Electronic Centre, Alexander Crum Brown Road, EH9 3FF, UK; 9 Easter Bush Pathology, The Royal (Dick) School of Veterinary Studies and The Roslin Institute, Easter Bush Campus, Midlothian, EH25 9RG, UK; 10 MRC Centre for Inflammation Research, The Queen’s Medical Research Institute, University of Edinburgh, Edinburgh EH16 4TJ, UK

## Abstract

Dysfunction of cell-cell tight junction (TJ) adhesions is a major feature in the pathogenesis of various diseases. Liver TJs preserve cellular polarity by delimiting functional bile-canalicular structures, forming the blood-biliary barrier. In acetaminophen-hepatotoxicity, the mechanism by which tissue cohesion and polarity are affected remains unclear. Here, we demonstrate that acetaminophen, even at low-dose, disrupts the integrity of TJ and cell-matrix adhesions, with indicators of cellular stress with liver injury in the human hepatic HepaRG cell line, and primary hepatocytes. In mouse liver, at human-equivalence (therapeutic) doses, dose-dependent loss of intercellular hepatic TJ-associated ZO-1 protein expression was evident with progressive clinical signs of liver injury. Temporal, dose-dependent and specific disruption of the TJ-associated ZO-1 and cytoskeletal-F-actin proteins, correlated with modulation of hepatic ultrastructure. Real-time impedance biosensing verified *in vitro* early, dose-dependent quantitative decreases in TJ and cell-substrate adhesions. Whereas treatment with NAPQI, the reactive metabolite of acetaminophen, or the PKCα-activator and TJ-disruptor phorbol-12-myristate-13-acetate, similarly reduced TJ integrity, which may implicate oxidative stress and the PKC pathway in TJ destabilization. These findings are relevant to the clinical presentation of acetaminophen-hepatotoxicity and may inform future mechanistic studies to identify specific molecular targets and pathways that may be altered in acetaminophen-induced hepatic depolarization.

Hepatic TJs are cell-cell adhesions that preserve cellular polarity by delimiting functional bile canaliculi structures, forming the blood-biliary barrier. TJ barrier dysfunction is implicated in hepatitis, primary biliary cirrhosis and cancer; as well as inflammatory bowel disease^1^. Chemical disruption of adhesion sites can also have profound effects. Cholestatics (chlorpromazine; cyclosporine A) destabilize intercellular TJs via reactive oxygen species-mediated effects on TJ-associated F-actin distribution in human hepatic HepaRG cells^2^,^3^, whilst the tumour-promoter, phorbol ester, a specific protein kinase C-alpha (PKCα) activator, reduces TJ integrity and ablates cell polarity in HepG2 cells^4^,^5^. Indeed, pro-apoptotic signals are imparted by diminished intercellular and cell-substrate interactions. Previously, we demonstrated that serum from patients with acetaminophen (APAP)-induced acute liver failure (ALF), when applied to primary human hepatocytes (PHHs), cause a loss of cellular adhesion, hepatocyte detachment with actin-cytoskeletal disruption preceding apoptosis, via a β1-integrin pathway^6^,^7^.

Although APAP toxicity remains the leading cause of acute liver failure, temporal and quantitative effects of direct APAP toxicity on tight junction/adhesion structures have not been previously explored. Well-described biochemical endpoint assays reveal active and complex intrinsic pathophysiological mechanisms. APAP toxicity is caused by formation of the highly reactive metabolite N-acetyl-p-benzo-quinoneimine (NAPQI), via cytochrome P450 metabolism; followed by glutathione and ATP depletion, mitochondrial dysfunction, and oxidative stress; which culminates in necrotic cell death through physical disruption of cellular integrity^8^ and liver cytoarchitecture (centrilobular necrosis)^9^.

Normal hepatic tissue architecture is maintained by establishment of functional hepatic polarity through stable cell-cell/cell-matrix adhesions, which have key roles in signal transduction pathways regulating hepatic differentiation status. Recent insights demonstrate the importance of intact intercellular and cell-substrate interactions. Hepatic TJs maintain structural polarity essential for bile secretion, drug transporters and CYP450 expression^10^. Integrin-mediated cell adhesion sites link the actin-cytoskeleton with the extracellular matrix, and serve to modulate many aspects of cell behavior including survival/apoptosis, differentiation, and polarity^11^,^12^. Intercellular junctions are comprised also of anchoring junctions and gap junctions, also important in cell polarity and communication, including transport of reactive oxygen species^13^. Indeed, recent studies in mice implicate hepatic gap junctions as essential mediators of APAP-induced drug-induced liver injury (DILI)^14^,^15^,^16^, whilst gap-junction and TJ protein expression are closely correlated in hepatocytes and both are associated with actin filaments^17^.

PHHs represent the most pertinent *in vitro* hepatotoxicity model, notwithstanding their inherent limitations including: short culture life-span, phenotypic variability and instability in culture, with intermittent supply and high unit costs^18^. Recent studies highlight the need to develop alternative cell systems to PHHs for initial hepatotoxicity screening or mechanistic studies of model hepatotoxins or candidate compounds^19^.

HepaRGs, a bipotent human hepatic cell line, are increasingly regarded as a surrogate to PHHs, providing an excellent model system for exploring mechanisms of APAP hepatotoxicity and cellular polarity^18^,^20^,^21^. HepaRG cells form a stable (4 weeks) hepatic co-culture, of hepatocyte-/and cholangiocyte-like cells with highly differentiated, functional morphology. They provide a physiologically-relevant *in vitro* liver model, with intact drug metabolism, and functional polarity with bile canalicular structures, delineated by junctional complexes.

Mechanisms of APAP hepatotoxicity are reported to be similar in both humans and mice. Indeed, clinically-relevant mechanisms of APAP toxicity in human hepatic HepaRG cells are consistent with mouse studies of APAP hepatotoxicity^20^,^22^. Whereas, a recent toxicogenomics study, shows that HepaRG cells have the highest predictive capacity for APAP-induced ALF, compared with PHHs, HepG2 or induced pluripotent stem cell derived hepatocyte-like cells^23^.

Current methods to assess cell-cell, and cell-substrate adhesions, involve mostly well-defined end-point assays such as: immunostaining, ultrastructural imaging, qRT-PCR, Western blot or flow cytometry techniques. In addition, methods to measure transepithelial electrical resistance (TER), a measurement of cell barrier function established by TJs formation, are available, although rarely utilized in liver cell biology. Macromolecular permeability assays remain a straightforward and well-established technique for studying (endothelial) barrier function. They are easy and robust, but not very sensitive towards rapid and transient changes^24^. In this study, we used Electric cell-substrate impedance sensing (ECIS) to examine the effect of APAP on the liver barrier function in real-time. In addition, we wanted to assess the cell-substrate adhesion parameter and correlate this with flow cytometry data (integrin expression), as well as the cell membrane capacitance as a marker of membrane integrity. These parameters can only be obtained using the ECIS platform and cannot be derived from transwell macromolecular assays or from other impedance sensing equipment^24^,^25^.

Electric Cell-substrate Impedance Sensing (ECIS) provides real-time, non-invasive, quantitative measurements of parameters related to cell behaviour. By exploiting impedance-spectral modelling using ECIS, unbiased assessment of cellular response, including barrier function and cell-substrate adhesion, is possible^26^. Consequently, throughout this study, HepaRG and ECIS microelectrodes were combined to provide unique insights into temporal dose-dependent disruption of intercellular and cell-substrate adhesions in response to the model hepatotoxin, APAP. In this study, our aim was to explore the possible effects of APAP on the stability, phenotype and distribution of hepatic TJ-associated structures in human hepatocytes, and to investigate this further *in vivo* in a murine model of APAP-induced liver injury.

Herein, we demonstrate a feature of APAP hepatotoxicity not previously described; temporal and progressive effects on liver cell cohesion and polarity through disruption of intercellular (tight) junctions and cell–substrate adhesions. Phenotypic profiling of human liver models *in vitro* demonstrated disruption of the TJ-associated structures ZO-1/F-actin and in hepatic ultrastructure. Real-time, quantitative impedance biosensing supported these observations, revealing early TJ-disruption (<3 hours) even at the lowest dose tested (5 mM APAP). At this dose conventional endpoint assay assessing ATP depletion did not detect cell necrosis, however, alternative assays demonstrated enhanced oxidative stress, reduced metabolic competence and membrane integrity (Cm impedance parameter), accompanying TJ perturbation. To validate the *in vitro* observations, we demonstrated in a mouse model of APAP-induced liver injury, a progressive, dose-dependent loss of intercellular hepatic (tight) junction ZO-1 expression with enhanced liver damage evident at low-end dose (150 mg/kg APAP). TJ-disruption occurred in mouse liver at the lowest dose 75 mg/kg APAP, in the absence of classic indicators of drug-induced liver injury (liver ALT elevation). Given TJs are emerging as an important cellular signaling hub and may act as sensors for tissue stress and damage^5^,^27^, our observations may prime future mechanistic studies to unravel complex TJ signaling pathways involved in APAP-induced stress.

## Results

### Effects of APAP on structural determinants of epithelial polarity in HepaRG cells

#### APAP effects on cytoskeletal F-actin stability

**Figure 1:**
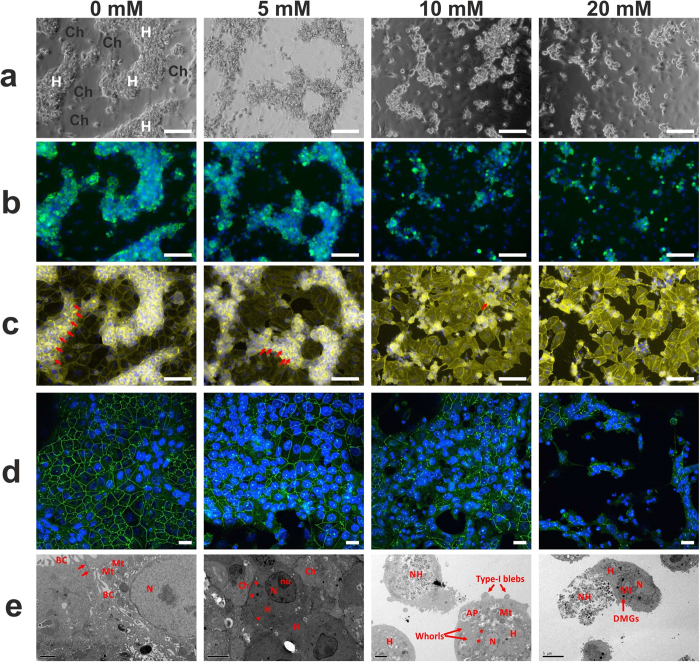
Disruption of hepatic architecture in HepaRG cells following 24 hours APAP treatment (a–c). Characteristic hepatocyte (H)/cholangiocyte (Ch) *in vivo*-like hepatic cord phenotype in control cultures is progressively lost with increasing APAP concentration. This is demonstrated in phase-contrast images (**a**), with (**b**) Transferrin co-localization (green; Hoechst nuclear counter-stain, blue), a highly-specific marker of mature hepatocytes, and (**c**) Strong F-actin/Phalloidin staining (yellow) reveals distinct pericanalicular-cytoskeletal/TJ-associated F-actin bands (red arrows) in control cells and with decreased staining at low-dose (5 mM) APAP (arrowheads); and progressively less intense staining at higher APAP dose; consistent with a direct effect of APAP/and or APAP reactive metabolites on actin structures. (**d**) Confocal microscopy demonstrated reduced intensity (green immunofluorescence staining) of the hepatic tight junction-associated structural protein ZO-1, with increasing APAP. The ‘chicken wire’ network of delineated by ZO-1, progressively decreases, as the hepatic cord phenotype is compromised. (**e**) Representative TEM images: Left panel shows typical ultrastructure in untreated controls, showing numerous mitochondria (Mt) with tight junctions (arrows), which seal bile canalicular lumen (BC) formed between two adjacent hepatocytes. At 5 mM APAP, although discrete TJ structures were not visible, an ‘electron-dense’ perimeter surrounded hepatocytes (red arrowheads), adjacent cholangiocytes (Ch) are shown. At 10–20 mM APAP, TJs were not present, with cells commonly exhibiting necrotic (NH) or apoptotic appearance, with Type-I blebbing, and pseudomembranous structures (whorls) representing autophagolysomes; altered mitochondria shape was evident, although retaining some dense mitochondrial granules (DMGs). Scale bars: 100 μm (**a–c**); 50 μm (**d**); and as indicated in TEM micrographs. Nucleus (N), nucleolus (nu).

Since hepatotoxins can destabilize intercellular TJs via TJ-associated F-actin disruption, we monitored phenotypic changes in cytoskeletal F-actin in response to APAP-treated HepaRG cells. Phase contrast imaging shows, in HepaRG cells, typical ‘hepatocyte island’ phenotype with cholangiocyte-like (H, and Ch, respectively, in Fig. 1a) morphology in untreated control (0 mM) (Fig. 1a). In Fig. 1b, both untreated cells and cells treated with 5 mM APAP, exhibited strong expression of hepatocyte-specific Transferrin in regions of hepatocyte-like ‘cords’, with punctate staining of circumferential F-actin bands (Fig. 1c), indicative of bile-canalicular structures. On treatment with APAP we observed a dose-dependent reduction in F-actin/phalloidin staining (Fig. 1c), indicating destabilization of TJs.

#### Loss of HepaRG intercellular/cell-substrate adhesion integrity following APAP toxicity

Next, we performed ultrastructural imaging and ZO-1 (TJ-associated)/E-cadherin immunofluorescent staining after exposure to APAP, from subtoxic to toxic levels. Confocal microscopy demonstrated a dose-dependent structural disruption of both TJ, as assessed by reduced ZO-1 fluorescent-staining (Fig. 1d), and expression of the junctional-associated protein, E-cadherin (Supplementary Fig. 7). TEM analysis showed characteristic normal hepatic ultrastructure^28^ in HepaRG controls (0 mM APAP), including intact TJ and bile canaliculi, confirmation of hepatic polarity. Dose-dependent disruption of hepatic architecture (Fig. 1e) was evident with TEM, even at low-dose (5 mM) APAP, prior to cell death (type 2 blebbing) ≥10 mM. Examination of ultrastructure at 5 mM APAP showed no evidence of discrete TJ structures (Fig. 1e). However, we did observe an ‘electron-dense’ perimeter around the hepatocytes; indicative of subcellular redistribution of TJ proteins in response to cellular stress, contributing to destabilization of the epithelial barrier^28^.

#### Establishment and characterization of HepaRG-based impedance toxicity assay

**Figure 2:**
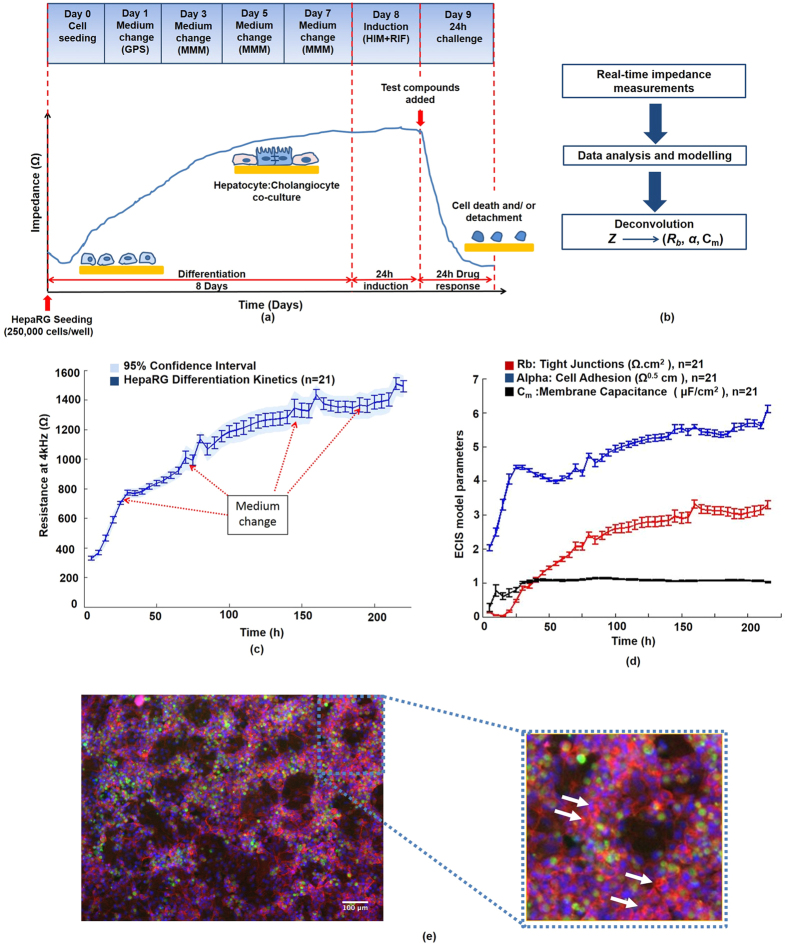
Establishment and characterization of human hepatic HepaRG-based model on impedance sensing arrays. (**a**) Technical workflow for the human HepaRG-based liver-on-a-chip approach: HepaRG cells are seeded at high density on microelectrode arrays. The impedance, Z, is recorded in real-time throughout cell differentiation (8 days). HepaRG cells self-organize into a terminally-differentiated hepatocyte:cholangiocyte co-culture; which was mirrored by an increase in resistance at 4 kHz, indicative of tissue barrier formation. After induction, a 24 h time- and dose-response is recorded at multiple frequencies. Impedance values of cells exposed to highly toxic drug levels, can reach the value of the cell-free electrode, demonstrating complete cell death and detachment from the microelectrodes. But critically, early and low-dose effects are mainly found to disrupt hepatic adhesion structures. (**b**) The different current pathways are analysed using the built-in ECIS model. The impedance is then deconvolved into biologically-relevant cell electrical parameters: Rb (cell-cell junctions), z-alpha (cell-electrode adhesion) and Cm (cell membrane capacitance). **(c)** HepaRG differentiation kinetics: As the cell population undergoes self-organization/maturation, the resistance at 4 kHz increased steadily until reaching a plateau on day 7, reflecting completion of the differentiation/maturation phase, and epithelial polarization; as evidenced by: (**d)** retrieval of parameters related to barrier function (Rb), cell-substrate adhesion (z-alpha) and cell membrane capacitance (Cm)-confirming establishment of HepaRG cell-cell tight junctions, cell-electrode adhesion and stable, intact cellular membranes (Cm). (**e)** HepaRG cells exhibited a highly differentiated phenotype (day 8): Tri-colour fluorescent-staining revealed extensive hepatic CYP3A4 enzyme activity (green); punctate staining of F-actin bands, indicative of bile-canalicular structures (red; Phalloidin-staining; white arrows); with *in vivo*-like hepatic cords (H) and cholangiocyte-like cells (Ch; ‘voids’).

The ability of the impedance-based HepaRG system to report on toxicity-induced disruption of cell-cell junctions and cell-substrate adhesions depends on the establishment of confluent liver-like cultures on top of the ECIS microelectrodes (Fig. 2). Terminally-differentiated HepaRG cells were allowed to self-organize for 8 days until they formed *in vivo*-like hepatic cords and cholangiocyte-like cells with functional polarity, with high CYP3A4 activity (Fig. 2e; green-staining) indicating metabolic competence for APAP metabolism (Fig. 2e). At toxic levels of APAP, the CYP450s convert APAP into the reactive metabolite NAPQI^8^. Importantly, kinetics of HepaRG growth, spreading, differentiation and barrier function formation, were monitored in real-time (Fig. 2b,c), prior to toxicity tests; defining, *de facto* quantitative criteria of performance, thus improving assay robustness. This was achieved by collecting the total impedance signal (**Z′**) at multiple frequencies [62.5–64,000 Hz], from which three parameters: Rb (**Ω.cm**^**2**^); z-alpha **(Ω**^**0.5**^**cm)** and Cm **(μF/cm**^**2**^); respectively, associated with cell-cell tight junctions, cell-substrate adhesion, and membrane integrity, were extracted (Fig. 2; Supplementary Fig. 1). The requirement for total microelectrode coverage (cell confluence) prior to impedance toxicity assay, was confirmed by microscopy (see Fig. 1a,b; Fig. 2d). Growth kinetics and establishment of TJs in PHHs are shown in Supplementary Fig. 9.

### Impedance (ECIS) measurements: Time- and dose-dependent response of HepaRG cells to APAP (0–24 hours)

#### Modulation in overall (global) impedance signal

**Figure 3:**
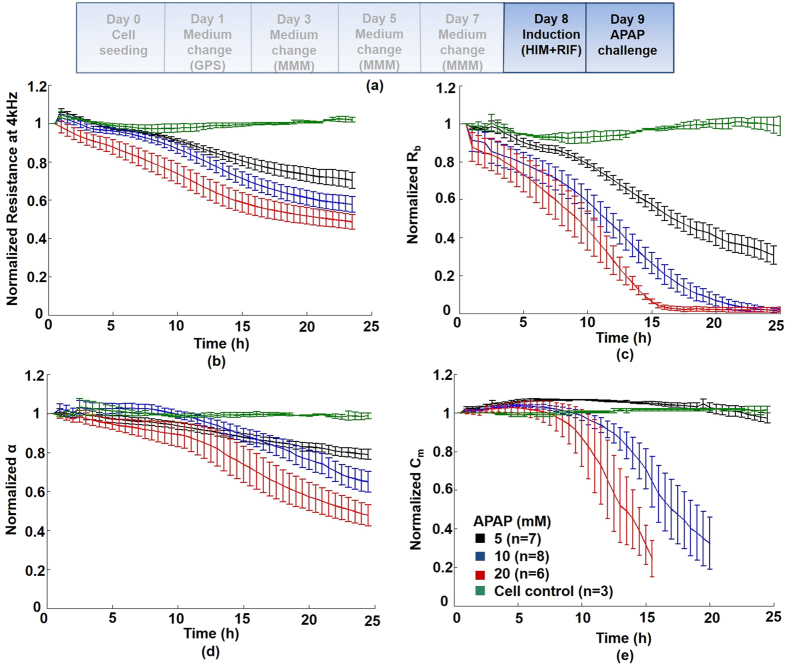
Real-time impedance monitoring of HepaRG-based liver-on-chip device following 24 hour APAP challenge. **(a)** Protocol time line: Following 24 hours rifampicin induction in confluent HepaRG cells, cells were treated for 24 hours with APAP. (inset key: untreated cell control; 5 mM; 10 mM; and 20 mM). (**b**) Post-challenge resistance kinetics: APAP caused a dose-dependent decline in normalized resistance - a global indicator of cellular status. (**c**) Rb (cell-cell tight junctions): APAP disrupted TJs in a concentration- and time-dependent manner; compared with control values, Rb decreased significantly (by 6 hours) at 10 and 20 mM APAP (P < 0.01; Supplementary Table 1). (**d**) z-alpha: Cell-substrate adhesion disruption was detected at <10 hours, suggesting loosening of cells from the electrode surface. At 5 mM APAP, cell adhesiveness decreased 10% at 6 hours, to 25% by 12 hours, and below 50% at 24 hours (P < 0.01; Supplementary Table 1). No change in cell behaviour was observed with standard biochemical toxicity assays at this dose (Supplementary Fig. 2). (**e**) Quantitative Cm values (membrane capacitance), reflecting cell membrane integrity, were significantly compromised both at high APAP doses (10–20 mM), and earlier (12 hours), than those of cell viability measured in corresponding 24 hour biochemical toxicity assays. See Supplementary Table 1 and Supplementary Fig. 3 for corresponding non-normalized data.

Figure 3a shows the protocol time line (described in Online Methods). APAP induced a time- and dose-dependent decrease of impedance at all frequencies, indicating a global decline in cellular health over 24 hours (Fig. 3b). Quantitative impedance measurements were acquired every 160 s over 24 hours, in an effort to detect earlier, key biological events (cell behavior parameters: Rb + z-alpha + Cm) in response to APAP. Figure 3b demonstrates the corresponding decrease in ‘global’ resistance at 4 kHz, approaching the value of cell-free electrodes at the highest dose (20 mM) at 24 hours; as shown in non-normalised data (Supplementary Fig. 3; Supplementary Table 1).

#### APAP disruption of tight junction structures (Rb)

In order to investigate the effect of APAP metabolites on TJs, we first performed a 24 hour treatment with rifampicin (CYP3A4 inducer); during this period minimal effects on both global impedance or the above modeled parameters of cell behavior were observed (Supplementary Fig. 6). We then applied APAP to the cells for 24 hours, where we detected significant, time-/dose-dependent decreases in the normalized cell-cell TJ parameter, Rb (Fig. 3c). Non-normalised data, showing absolute impedance values with statistical significance, is shown in Supplementary Table 1 and Supplementary Fig. 3. Compromised TJs appeared rapidly (<3 hours), even after treatment with low levels of APAP (5 mM), whilst Rb was completey abolished at 20 mM APAP (toxic levels), after just 15 hours.

#### Hepatotoxicity in HepaRG cells following APAP

Cell-mediated cytotoxicity was further confirmed at 24 hours (Supplementary Fig. 2), by multiplexing live-cell viability (PrestoBlue), with classic ATP-depletion endpoint (CellTiter-Glo) assays. These conventional assays, estimate the functional or metabolic state of the cell, typically, mitochondrial reductive activity/dysfunction, as a correlate of oxidative stress in response to a hepatotoxin. Notably, at a low dose of 5 mM APAP, the ATP-depletion assay did not reflect significant disruption to TJ or cell-substrate adhesions as observed by impedance measurements ≤24 hours (Fig. 3c,d). However, Prestoblue, a live viability assay, demonstrated impaired metabolic activity at 5 mM APAP in HepaRG cells (SI Fig. 2a); whereas ATP-depletion endpoint assay (SI Fig. 2a) only detected cell stress at higher APAP doses (10, 20 mM APAP). Finally, we demonstrated similar effects of APAP on PHHs including TJ disruption (Supplementary Information Fig. 8), although with little effect on hepatotoxicity parameters (Supplementary Information Fig. 10). The latter observation may be related to batch variation of PHHs, culture conditions (±collagen) and perhaps the mode of cell death for which they may be primed (higher ATP content in pre-apoptotic cells)^19^.

#### Markers of cellular stress and DILI in HepaRG cells following APAP: Lactate and Albumin production

We measured lactate production, as an alternative oxidative stress biomarker for DILI^31^,^32^ in HepaRG cells following challenge with APAP (0–40 mM) for 24 h. Both lactate production increased, whilst albumin declined significantly after exposure to APAP at low dose (5 mM; p < 0,05) – and with increasing doses of APAP (Supplementary Fig. 11). This demonstrates a significant pathophysiological response accompanying TJ-disruption, given these are considered sensitive markers for detecting drug-induced hepatotoxicity *in vitro*^31^,^32^.

### Chemically-induced disruption of cell-cell adhesion structures

**Figure 4:**
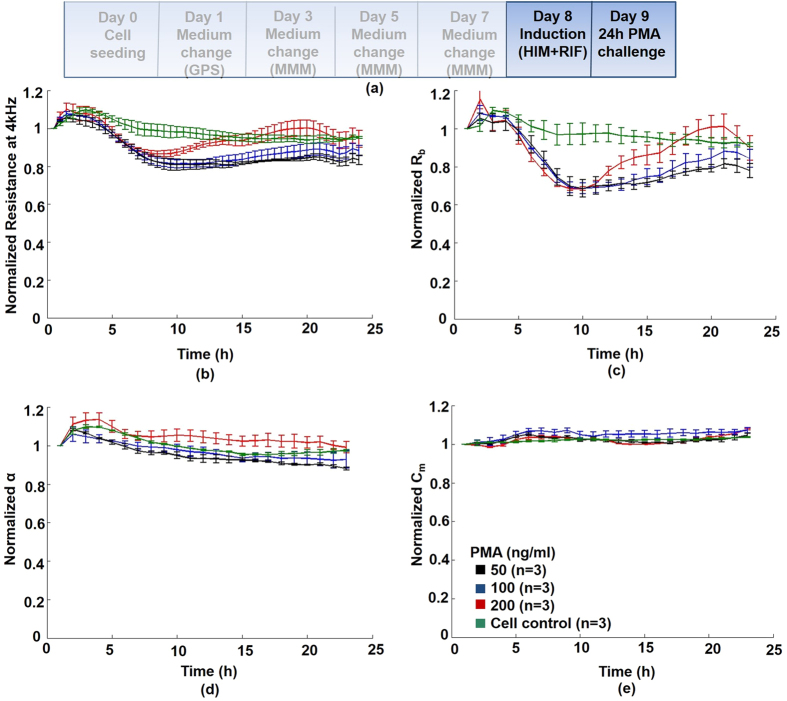
HepaRG tight junction disruption: Real-time impedance monitoring following 24 hours treatment with the TJ-disruptor phorbol-12-myristate-13-acetate (PMA). (**a**) Protocol time line: HepaRG cells were treated as described in online methods. Initially, normalized resistance (global indicator of cellular status) decreased significantly (P < 0.01) by 20%, (4–10 hours), compared with untreated control cells, followed by a return to pre-challenge resistance values; (**b**) Rb (cell-cell tight junctions): the only quantified effect of the PKC-activator PMA was on Rb, which decreased at all doses tested (6–12 hours), and significantly at intermediate (100 ng/ml; P < 0.05)/high dose (100 ng/ml; P < 0.01) PMA (Supplementary Table 3; Supplementary Fig. 4) - returning close to original (control) values (between 10–22 hours); (**c,d**) Minimal effects of PMA were detected on either cell-electrode adhesion (z-alpha), or Cm (cell membrane integrity). Parallel hepatotoxicity assays were also performed (Supplementary Fig. 2c,d).

**Tight junction disruption via phorbol ester activation of protein kinase C (PKC):** Phorbol ester has been shown to abrogate TJ integrity and significantly reduce polarity in human hepatic HepG2 cells^4^. To validate quantitative Rb (TJ) impedance measurements, and further test if APAP-induced disruption of TJs could implicate a classic PKC-dependent signalling pathway, we performed a dose-response with the PKC-activator, phorbol-12-myristate-13-acetate (PMA) (Fig. 4). The main effect of PMA was quantified on normalized Rb (see also Supplementary Table 3; Supplementary Fig. 4), showing a ≈30% decrease (*vs* control), at all doses (4–10 hours), and returning close to original (control) values after several hours (Fig. 4c), possibly due to detoxification of PMA by ubiquitous liver Phase I carboxylesterases. A slight elevation in cell viability and ATP, was observed at 24 hours (Supplementary Fig. 2), this could be due to hormetic effects of PMA, whereby hepatocytes can respond to toxic stimuli through proliferation and metabolic activation. Similarly, a transient increase in impedance was observed during the first 3 hours, in all PMA concentrations tested (Fig. 4b).

#### Direct effects of NAPQI on tight junctions

NAPQI formation is a major mediator of APAP hepatotoxicity, we therefore tested direct effects of different ‘bolus’ concentrations of NAPQI on TJs (Rb parameter) with impedance sensing (Supplementary Fig. 5). The doses tested (0–500 μM) are supra-physiological, since dosing of purified NAPQI < 500uM, does not elicit any response *in vitro*^33^. At a high dose of NAPQI (500 μM), non-normalized resistance showed a sustained decrease over 20 hours. An abrupt (0–2 hours) and sustained (0–20 hours) disruption of TJs occurred, with a 50% decrease in the impedance parameter, Rb after 6 hours. Minimal effects on Rb at 125 and 250 μM, on both Rb and z-alpha, may reflect detoxification of NAPQI by intrinsic stores of glutathione, as high doses of APAP are required to deplete intracellular glutathione. Previous studies in rat hepatocytes, suggest NAPQI has an extremely short half-life (2 minutes), with latent effects on cell integrity occuring between 10–120 minutes, using similar NAPQI concentrations 100–500 μM^34^,^35^.

#### APAP disruption of cell substrate adhesion structures (z-alpha)

We observed both a time- and dose-dependent reduction in the cell-adhesion impedance parameter (z-alpha) over 24 hours APAP exposure (Fig. 3d). Compared with untreated cell controls, disruption to cell adhesion, although not statistically significant, was detected at <10 hours (Supplementary Table 1). At 12 and 24 hour time points, z-alpha could clearly quantify and discriminate, significant, early time-/dose-dependant effects of APAP on cell adhesion in real-time (Supplementary Table 1); in contrast with conventional techniques, (24 hour end-point hepatotoxicity assays; Supplementary Fig. 2). Of note, even at low levels of APAP (5 mM), we observed significant decrease in cell adhesion at both 12 and 24 hours (Supplementary Table 1).

#### Flow cytometric integrin expression of attached HepaRG cells following 24 hours APAP exposure

**Figure 5:**
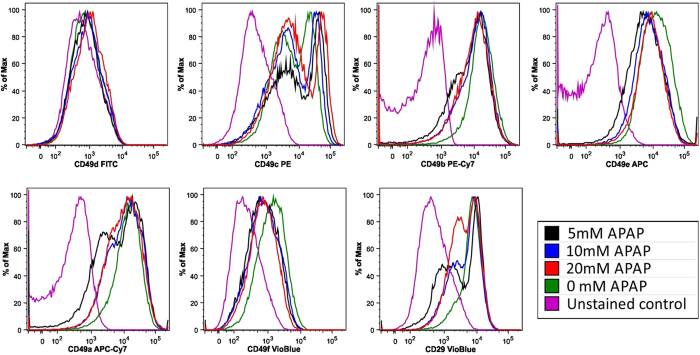
Flow cytometry analysis of integrin expression in HepaRG cells following APAP treatment for 24 hours. Histograms representing relative expression of integrins (CD49a-f) and CD29 in HepaRG cells following challenge with different concentrations of APAP. Though the expression of most integrins was modulated over the period of exposure to APAP, only modulation of CD49c was directly associated with increasing APAP concentration. Untreated HepaRG cells contained high and low CD49c expressing populations. 24 hours after treatment, increasing APAP concentration was directly associated with increased percentage of cells in the low expressing population, and a reciprocal decrease in the high expressing population. The level of expression (MFI) was unchanged in the CD49c low population, whereas the level of expression by cells in the high population increased directly with APAP concentration. Detailed flow cytometric data is shown in Supplementary Table 2.

We performed flow cytometric analysis to assess the association of z-alpha with local modulation of cell adhesion in response to APAP hepatotoxicity. In the liver, the effect of model hepatotoxins such as APAP on integrins, remains poorly understood. To this end, we screened HepaRG cells against a panel of monoclonal antibodies (Supplementary Table 2), to identify surface molecules that are modulated on exposure to APAP. We identified a number of candidates that were expressed in untreated HepaRG cells; CD29 (β1 integrin) and both CD49a-c, and CD49e-f (α integrins 1–3, and 5–6, respectively) (Fig. 5) - in agreement with previous studies^36^,^37^. An increased percentage of cells in the low CD49c expressing population was directly associated with increasing APAP concentration. In addition, low-expressing sub-populations of CD29, CD49a and CD49b were detected, but did not appear to be directly associated with APAP concentration. Increasing APAP concentration resulted in an increased percentage of CD49c low expressing cells with little change in mean fluorescent intensity (MFI); whereas the observed CD49c high population MFI increased directly with APAP concentration.

### Chemically-induced disruption of cellular integrity

#### APAP disruption of cellular integrity (Cm)

In contrast to Rb and z-alpha, cell membrane capacitance (Cm), a measure of cellular integrity, was compromised only at higher doses of APAP (10 and 20 mM), with minimal effects observed at low dose (5 mM) (Fig. 3e). However, onset of a significant and abrupt drop (≈70% *vs* control) in Cm was detected at 8 hours with 20 mM APAP, and 12 h with 10 mM APAP, indicative of sudden rupture of the cell plasma membrane. Thus, onset to major cell membrane disruption, for the majority of cells exposed to APAP (quantified at 15 hours, 20 mM; and 20 hours, 10 mM; see Supplementary Table 1), could be identified earlier than by standard biochemical assays (Supplementary Fig. 2).

### Expression of ZO-1 following APAP-induced murine liver injury *in vivo*

#### Histopathology and immunohistochemical quantification

**Figure 6:**
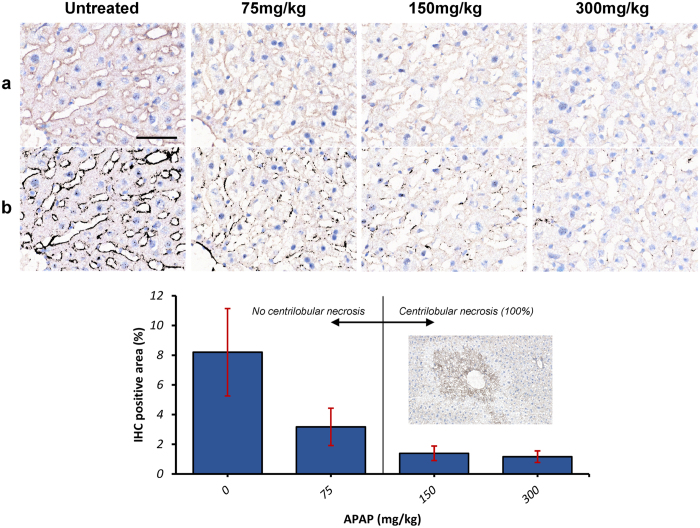
Hepatic Expression of ZO-1 following treatment of mice with increasing doses of APAP: Histopathology and immunohistochemical quantification. ZO1 immunohistochemical staining highlights hepatocytes and is progressively lost with increasing APAP dose (0, untreated; 75–300 mg/kg APAP; n = 2 animals per treatment group). Raw pictures (pre-processed) are displayed in (**a**), and the results of the DAB detection algorithm in (**b**). Note the membranous, continuous ZO-1 staining highlighting hepatic cords/sinusoidal areas in a control liver, and the progressive loss of ZO-1 staining with increasing APAP dose. ZO-1 staining becomes intermittent at 75 mg/kg, and virtually non-existent at 300 mg/kg. The bar chart in (**c**) displays the results of the IHC detection algorithm, which parallel those of the images in **a** and **b**; Inset: section of mouse liver showing typical APAP-induced centrilobular necrosis, at intermediate (150 mg/kg) and high (300 mg/kg) APAP dose, but not at low dose (75 mg/kg). IHC, DAB Haematoxylin, scale bar = 50 μm.

To assess whether TJ-destabilization occurs *in* vivo, we analyzed ZO-1 expression in livers of mice treated with low-high dose APAP [75–300 mg/kg]. Diagnostic liver function tests were performed (LFTs) to analyse blood serum enzymes to assess liver damage or cytosolic enzyme leakage. In control (untreated murine livers), ZO-1 expression characterized by IHC exhibited mostly continuous, membranous staining that outlined hepatocyte cords (Fig. 6a,b). This was noted in all areas (centrilobular, midzonal, and periportal), as well as around the centrilobular vein, with normal LFTs (Aspartate Amino Transferases; ASTs ≤ 268 U/L). This pattern of expression was progressively lost, in a dose dependent manner, with increased discontinuity of membranous ZO-1 staining at low-dose [75 mg/kg] APAP, which progressed to virtually absent ZO-1 staining at 300 mg/kg (Kruskal Wallis test, p = 0.04; Fig. 6c).

### Pathophysiology following APAP-induced murine liver injury *in vivo*: Liver Function Tests

Characteristic centrilobular hepatic necrosis combined with highly elevated LFTs (*range*) was observed only in mice treated with 150 mg/kg (ALTs: 700–12820 U/L; ASTs: 550–16560 U/L; n = 6 animals) and 300 mg/kg APAP (ALTs: 2150–8980 U/L; ASTs: 2550–10420 U/L; n = 5 animals). LFTs were at control levels for mice treated with low-dose 75 mg/kg APAP (eg serum ALT was normal, 32–72 U/L). However, increased Bilirubin and ALP was also observed in mice treated with 150 and 300 mg/kg APAP, hallmarks of acetaminophen-induced liver injury (AILI) with cholestasis. Taken together, these data suggest both specificity of the effects of APAP on the tight junction apparatus (ZO-1) with accompanying liver pathology *in vivo*.

## Discussion

Despite the central role of hepatic TJs and cell-substrate adhesion structures in maintaining differentiated functional polarity and xenobiotic metabolic competence, there are no reported studies of APAP hepatotoxicity in this context. TJ destabilization, evident even at an APAP dose generally considered sub-toxic (5 mM), was confirmed via disruption of the TJ-specific protein ZO-1 and through ultrastructural imaging of HepaRG cells (Fig. 1); which in turn, demonstrated phenotypic changes indicative of apoptotic (cellular Type-I blebbing) and necrotic cells, at intermediate dose (10 mM). Bleb formation is an early marker of actin-cytoskeleton disruption, whilst redistribution of ZO-1, occurs as a consequence. The TJ adaptor proteins ZO-1 and ZO-2, are anchored to the actin cytoskeleton, indicating a potential role for TJs in the organization of the actin cytoskeleton^38^; therefore disruption could compromise ZO-1 mediated TJ stability. We previously demonstrated in human hepatocytes that serum from patients with APAP-induced liver failure caused actin-cytoskeletal disruption, loss of β1-integrin activity and cellular adhesion resulting in caspase-3–mediated apoptosis^6^. Taken together, given tight junctions are directly linked to the actin cytoskeleton, discriminating disruption of cell structural adhesions allows priming of subsequent investigations into toxicity-related molecular initiating events, and lends itself to assessing the potential role of actin disruption in cell death^39^.

Using confocal microscopy, we observed an APAP-dependent diminution in immunofluorescent staining of both the TJ-associated proteins ZO-1 (Fig. 1d) and E-cadherin (Supplementary Fig. 7), indicative of injury to the epithelial tight junction complex (at 24 hours), and, consequently, lateral polarized phenotype. This was coincident with an early (<6 hours), and sustained fall (<12 hours) in the real-time Rb impedance signal at all APAP concentrations (Fig. 3c; Supplementary Table 1). This highlights an advantage of our approach over existing invasive methods to detect perturbations of structural determinants of epithelial polarity. Dysregulation of these proteins may result in decreased cell survival, and activation of cell death pathways^7^, through disruption of hepatic TJ integrity and adhesion to extracellular matrix components. Increased Lactate with decreased albumin production, as markers of cellular stress with DILI, was evident at low-dose (5 mM) APAP in HepaRG cells, indicative of accompanying pathophysiology to TJ-disruption *in vitro*. Lactate production has been demonstrated to be a sensitive and global marker of cellular stress for DILI in HepaRG cells^31^, and is linked to mitochondrial dysfunction^32^. Although ATP levels remained at control levels in HepaRG cells treated with 5 mM APAP, consistent with our *in vitro* data, low levels of oxidative stress induced by the pro-oxidant tert-butylhydroperoxide (tBOOH)^40^, had no measurable effect on cellular viability, LDH release, or ATP content. Despite these low levels of oxidative stress, participation of Ca^2+^-dependent PKCs in actin disorganization and tight-junctional impairment was demonstrated, with bleb formation. Furthermore, tBOOH induced translocation of the Ca^2+^-dependent PKC isoform, PKCα, to the cell membrane, a phenomenon associated with activation of the PKC isoform. In turn, PKCa activation is known to promote TJ disassembly, whilst increase in intracellular Ca^2+^ concentration in hepatocytes is a known consequence of acetaminophen toxicity^41^. We show additionally, that PMA, a known TJ-disruptor through activation of PKCa, reduced barrier function (Rb component) in HepaRG cells (Fig. 4) analogous to APAP-induced reduction in Rb (Fig. 3).

As an *in vivo* correlate of our findings in human HepaRG cells, we demonstrated in a murine model of APAP-induced hepatotoxicity TJ-disruption (decreased ZO-1 staining) at all doses. Consistent with these data, increased blood-brain barrier permeability was demonstrated in a mouse model of APAP-induced acute liver failure, which may be due to the loss of the TJ-associated protein occludin^42^. The lowest dose APAP (75 mg/kg), with a human equivalence dose (HED) of 360 mg APAP (<one tablet)^43^, resulted in decreased ZO-1 staining. For comparison, an *in vivo* dose of 800 mg/kg APAP is recognized as 100% lethal in mice^44^. Interestingly, although this change in ZO-1 staining generally occurred in the absence of classic APAP-induced centrilobular necrosis, we observed exacerbated liver damage (elevated LFTs) in one mouse (cohort, n = 5) treated with the lowest dose 75 mg/kg APAP (data not shown), which might resemble the variability that naturally occurs not only in murine but also in human DILI^45^. Also at 24 h post-APAP, we observed characteristic centrilobular hepatic necrosis (Fig. 6) *combined* with highly elevated LFTs (ALT: 700–12820 U/L; n = 5) in mice treated with 150 mg/kg and 300 mg/kg APAP, with a 2–3x fold increase in Bilirubin and elevated Alkaline Phosphatase (ALP), hallmarks of AILI with cholestasis. Given cholestasis is indicative of TJ-disruption through increased paracellular permeability, we suggest low-dose APAP manifests pathophysiological features in this cohort of mice. At 150 mg/kg APAP, ALT levels were normal in one mouse with elevated ASTs and normal histology, which appears consistent with the heterogeneity observed in animals and human variability in reaction to APAP. In agreement with Hu *et al*.^46^, this pathology, observed at low-dose APAP in our study, may be particularly relevant in the context of potentiation of toxicity in ‘susceptibles’ with pre-existing liver disease/inflammatory stress, such as leaky TJs in NAFLD, or chronic low-dose ‘therapeutic’ APAP toxicity^45^,^46^,^47^,^48^.

Recent studies in mice given a “sub-lethal” dose (250 mg/kg) of APAP demonstrate an early course of liver injury^49^, with clear increases in necrotic area/liver damage (6–12 hours post-APAP), as well as cytokine/chemokine receptor levels and serum transaminase levels (ALT). The latter is in agreement with our study of progressive increase in ALT/AST levels in mouse at all doses tested (albeit at 24 h), with concomitant reduction in both the impedance barrier function Rb (Fig. 3) and ZO-1 expression observed (Fig. 6). Therefore, potential impact *in vivo* could be activation of innate immune cells, eg Kupffer cells, which can secrete a variety of pro-inflammatory cytokines and chemokines, leading to inflammation/leukocyte infiltration. This requires further study. Early, overt hepatic injury has been documented in mice (3–4 h) treated with 400 mg/kg APAP; these pathologic changes actually preceded the rise in ALT levels (at 4.5 h)^44^. This study also found histopathophysiological changes, at only 90 and 180 min, which also preceded the rise in the serum ALT levels. This suggests real-time detection of structural changes to liver architecture, such as TJs, using ECIS, may provide important clues as to disease etiology.

We hypothesize that ZO-1 expression may be altered due to translocation/protein-trafficking to the perimeter of the cell membrane, at least at low-dose APAP. This is evident at the ultrastructural level in HepaRG cells treated with low dose (5 mM) APAP (Fig. 1e). Progressive and significant reduction in ZO-1 expression was observed at higher APAP dosage (150 and 300 mg/kg), concomitant with classic hepatotoxic pathology, including centrilobular necrosis (Fig. 6c). Of significance, there was no evidence of discrete TJ structures (observed in untreated controls) – but strong evidence of both necrosis and apoptosis under TEM (Fig. 1e). Interestingly, recent work shows that CCl_4_-induced oxidative stress in rats, resulted in disassembly of TJ and depolarization of hepatocytes, through disruption of atypical-PKC complex formation, evident after only 2 hours of CCl_4_-leading to liver cholestasis and cirrhosis^50^. Indeed, given atypical-PKC regulates TJ assembly and epithelial cell polarity, it is feasible that APAP-induced oxidative stress may also lead to dysregulation of TJ-mediated cell-signaling and membrane trafficking^51^, particularly at a high-dose of APAP, resulting in cell death. We may also speculate that lower doses of APAP (Supplementary Fig. 7), where expression of both TJ-associated cytoskeletal F-actin and the signaling transducer E-cadherin is maintained, provides sufficient hepatocytic polarity^52^, to avoid both entry into cell death mode with retention of phenotypic (structural) stability. This demonstrates the utility of the HepaRG organotypic model, given they are an intrinsic hepatocyte:cholangiocyte co-culture. Moreover, translocation of ZO-1 from TJs to the plasma membrane is also observed within 2–3 h of *Clostridium difficile* toxin-A exposure in colonic epithelial T84 cells, in the presence of increased ROS^53^. Indeed, agonists of classic PKC isoforms can induce translocation of ZO-1 from the cytoplasm to the plasma membrane in MDCK kidney cells in low extracellular calcium, conditions which favor TJ-destabilization and reorganization of the actin cytoskeleton, whereas oxidative stress can induce ZO-1 translocation via a PKC-dependent pathway^54^.

TJ disruption is an emerging pathology in a variety of diseases^55^,^56^, including: altered TJ-associated ZO-1 protein localization in gut tissue, in both non-alcoholic fatty liver disease (NAFLD) and steatohepatitis; microbial pathogenesis^53^, such as TJ-mediated viral entry in hepatitis; cancer development and metastasis; cholestatic drug hepatoxicity^2^,^3^; and genetic-based (TJ-associated Claudin-1 mutations) liver disease (neonatal ichthyosis and sclerosing cholangitis syndrome). Disease-in-a-dish approaches could be implemented with impedance spectral modeling, utilizing induced pluripotent stem cell technologies^57^; to investigate cell adhesion disruption in such disease states.

Current limitations of impedance-based assays for hepatotoxicity studies include the reliance on a single parameter, Cell Index, as a global indicator of cellular status; and the lack of an appropriate surrogate for PHHs. Indeed, recent studies show Cell Index is not always associated with cytotoxic effects, and is also dependent on optimized cell models^58^. A key step in our approach was combining organotypic, functionally polarized human HepaRG cells with impedance spectroscopy. HepaRG cells exhibit an adult-like hepatic phenotype, and provide, more so than any other hepatic cell line currently available^18^, intact drug metabolism and differentiated functionality, comparable with PHHs. This HepaRG polarized phenotype was confirmed by measuring impedance parameters Rb, z-alpha, and Cm (Fig. 2c), with detection of bile canaliculi-associated F-actin bands, CYP3A4, and tight junctions (ZO-1) in control cells (Figs 1c and 2e) as correlates of epithelial polarization, over 8 days, prior to impedance cytotoxicity assays.

The ability of the spectroscopic impedance sensing system to deconvolve the impedance into three biologically relevant parameters (Fig. 1; Technical workflow) was central to this study. These parameters have been exploited in diverse applications, such as measurement of TJ permeability, utilizing blood-brain barrier endothelial cells, or gut *in vitro* models, in response to pathogens^59^. Similarly, studies in polarized kidney epithelial cells, have directly linked the z-alpha parameter to cell–substrate interaction, which exhibits modulation by the Ca^2+^-mobilizing agonist, bradykinin^60^. Furthermore, our group demonstrated that, Cm, cell membrane capacitance, could be used as a phenotypic parameter to monitor stem cell differentiation^61^.

Our observations on APAP-mediated cell adhesion disruption led to follow-up studies aimed at providing some mechanistic insight into APAP toxicity. We demonstrated that the HepaRG cells treated with the PKC-activator, PMA, induced disruption of TJs (Fig. 4c), similar to APAP in HepaRG cells (Fig. 3c). This suggests that APAP toxicity, via oxidative stress, may involve a PKC-dependent signalling pathway^62^. PKC involvement is also observed in other cell types, including bronchial epithelial cells^63^, and *in vivo* following carbon tetrachloride (CCl_4_)-induced oxidative stress in rat liver^50^. PKC signaling is also shown to play an important role in *Clostridium difficile* toxin A-mediated damage on TJ structure and functions in human colonic cells^53^. Although PMA had no effect on the cell-substrate adhesion parameter, z-alpha (Fig. 4d); in HepG2 cells, PMA has been shown to stabilize and, even enhance, integrin expression^64^.

Furthermore, a reduction in cytoskeletal F-actin (Fig. 1c), was observed with increasing APAP, a possible initiating event in TJ/cell adhesion destabilization. Supporting this, in rat hepatocyte couplets, oxidative stress induced hepatocellular F-actin-cytoskeleton rearrangement and TJ impairment (redistribution of the TJ-associated protein ZO-1) by a PKC-mediated Ca^2+^-dependent mechanism^29^. Recent studies have shown that at hepatotoxic APAP dose activation of the PKCα isoform, leading to cell death via c-Jun-N-terminal Kinase-dependent signaling (JNK)^62^, whilst APAP can cause higher classic PKCa and lower adenosine monophosphate-activated protein kinase (a survival signal) expression that induces JNK phosphorylation and cell death^9^ in an *in vivo* mouse model of APAP hepatotoxicity.

Indeed, it has been demonstrated that activation of the PKC signaling pathway affects epithelial barriers^34^, whilst PKC–mediated phosphorylation of focal adhesion proteins may be responsible for disruption of integrin-associated focal adhesions^65^.

In addition to PMA-mediated TJ effects, NAPQI, a highly reactive metabolite of APAP, reduced cellular tight junctions parameter (Rb) in HepaRG cells. This may indicate a role for oxidative stress in disruption of TJ integrity (since no effect was observed on z-alpha or Cm), possibly via F-actin disruption (a known target of oxidative stress in DILI), as a toxicity-initiating event. GSH is depleted by NAPQI and excess NAPQI causes oxidative stress and binds covalently to liver proteins, which have been located on plasma membrane of hepatocytes *in vivo*^66^. Given NAPQI has a very short half-life (2 minutes)^35^, further potentially more sensitive impedance-sensing experiments could be performed as data can be collected with high time-resolution (f = 10 Hz; 10 points/second). Since approximately 4% of a dose of APAP is converted to NAPQI *in vivo*^67^, measurement of NAPQI concentrations in cell culture following APAP dosing, may allow calculation of threshold NAPQI toxicity for a given dose of APAP, and whether APAP itself has direct effects on TJs. The latter could be further investigated by modulating toxicity using combinations of N-acetylcysteine and non-toxic regioisomers of APAP. Given multi-organ failure is a consequence of acute liver failure, the utilization of impedance sensing to assess the contribution of free radicals, such as NAPQI, in non-hepatic organ dysfunction, particularly with *in vitro* gut models (HT-29; caco-2 cells) could be employed, to assess APAP-induced TJ destabilization in leaky gut epithelia^56^.

We observed a concurrent decrease in the quantitative cell adhesion impedance parameter z-alpha, with modulated expression of integrin proteins, such as β1-integrin, associated with specific alpha-chains of the CD49 family, including dose-dependent effects on α3-integrin. Integrins are heterodimeric cell surface receptors composed of α and β subunits. Differentiated HepaRG cells express β1-integrins associated with CD49a (α1-integrin) on hepatocytes and CD49f (α6-integrin) associated with cholangiocytes^36^. We observed complex effects on integrin (and other cell surface markers, including stem cell markers) expression (Supplementary Table 2), although precisely how altered cell adhesion via integrin activation influence different pathways such as apoptosis, are not fully understood. However, given integrins are intimately associated with F-actin, a primary target of oxidative stress, it is therefore conceivable that APAP toxicity may lead to disruption of cell adhesion structures via integrin destabilization. Elucidating such molecular mechanisms may help inform subsequent investigations into toxicity-related molecular initiating events or mode of cell death. Follow-up studies could investigate cell surface marker expression in chronic APAP hepatotoxicity to determine a possible role in chronic hepatitis (inflammation) – potentially leading to fibrosis and cirrhosis^48^.

In conclusion, herein we demonstrate an undescribed feature of APAP: namely a temporal, dose-dependent disruption of intercellular tight junction/adhesion structures even at low dose, concomitant with liver pathophysiology. This observation may have relevance in the clinical syndrome of APAP-induced ALF and may inform future mechanistic studies. We envisage that HepaRG-based impedanced-based biosensing could provide new insights in diverse areas of liver research such as cholestasis, microbial pathogenesis, NAFLD or APAP hepatotoxicity studies. Future studies may elucidate molecular mechanisms by APAP which disrupts tissue cohesion and polarity, as well as investigate APAP-induced effects in non-hepatic target organs such as gut.

## Methods

### Cell Culture

HepaRG cells (HPR116-TA08; Cryopreserved HepaRG™ cells; Biopredic Int., Rennes, France) were cultured using specialized media, following the supplier’s protocols. Each medium was made up using William’s E Medium with GlutaMAX™ (Sigma), as the basal medium; with appropriate additives (ADD). Additional methodologies are described in Supplementary Information. Briefly, for each culture format indicated, HepaRG cells were seeded (day 0) at 2.4 × 10^5^/cm^2^ initially in Thaw, Seed and General Purpose HepaRG® medium (ADD670) on either 8-well ECIS microelectrode arrays (impedance measurements), Corning 96-well tissue culture plates (end-point hepatotoxicity assays) or Lab-Tek II chamber slides (immunocytochemistry). On day 3, medium was changed to HepaRG™ Maintenance and Metabolism Medium (MMM; ADD620), and HepaRGs cultured to confluence (terminally-differentiated hepatocyte:cholangiocyte co-culture); thereafter, medium was renewed every 2–3 days with MMM. Confluence on the electrodes is required prior to impedance toxicity assays, and for subsequent ECIS-based modeling (see Fig. 2; and Supplementary Fig. 1). On day 8, cells were washed twice with HBSS (Sigma-Aldrich, Poole, Dorset, UK), and following 24 hours CYP3A4-induction with Rifampicin in HepaRG™ Serum-free Induction Medium (ADD650), HepaRG cultures were subject to a dose-response challenge^20^,^30^,^68^ with the model hepatotoxin acetaminophen (0–20 mM; A7085, Sigma-Aldrich), in MMM, using DMSO-vehicle as controls [<0.1% DMSO final concentration in all experiments]. CYP3A4 metabolizes APAP to the reactive metabolite, NAPQI; therefore, Rifampicin induction was used to synchronize CYP450 enzymes since solvents such as DMSO, a possible constituent of proprietary culture medium, can inhibit these. For ECIS-based measurements, impedance was monitored with measurements taken at 160s intervals over a 500 Hz to 64 kHz frequency (f) range, for up to 96 h post-induction (described in detail below). We used the ECIS-Zθ built-in mathematical model, which scans impedance measurements through various frequencies to recognize current pathways, translating these into quantitative data on cell-cell junctions (Rb), cell-electrode adhesion (z-alpha) as well as the cell membrane capacitance (Cm).

### Methods overview

A technical workflow, outlining the HepaRG-based model, and subsequent impedance spectral data deconvolution into biologically-relevant parameters of cell behaviour, are shown in Fig. 2 and Supplementary Fig. 1. Parallel assessment of cellular morphology, phenotype, protein expression, alone, or in the context of APAP hepatotoxicity in HepaRG cells, were conducted independently, alongside each ECIS experiment (see Supplementary Information). Furthermore, effects of both phorbol ester (Protein Kinase C (PKC)-activator which abrogates hepatic TJs integrity) and purified NAPQI, on HepaRG TJs were investigated. We also report in Supplementary Information, additional methodological details related to: HepaRG and primary human hepatocytes (PHHs) cell culture on ECIS microelectrodes; hepatotoxicity assays; flow cytometry analysis; immunocytochemistry (*in vitro*) and immunohistochemistry (*murine model of APAP-induced liver injury); morphological and ultrastructural assessment; and impedance spectral modeling data analysis. *For immunohistochemistry of our murine model of APAP-induced liver injury, it is well recognized that dosing of 300–400 mg/kg in mouse may cause significant acute liver injury, and is used to model human APAP overdose^22^,^62^.

Immunostaining and immunofluorescence microscopy. HepaRG cells grown on chamber slides (Nunc Lab-Tek II; Thermo Scientific) for 8 days, were exposed to APAP (0, 5, 10 and 20 mM) as described above. Depending on antibodies used, the cells were treated by fixation with 4% buffered formaldehyde solution (100469; Merck-Millipore). The cells were subsequently permeabilised with 0.25% Triton-X 100 (X100; Sigma-Aldrich) in Tris-buffered Saline (TBS; T6664; Sigma-Aldrich), and blocked with 5% goat serum (G6767; Sigma-Aldrich) in TBS, before they were serially exposed to appropriate primary and secondary antibody combinations (See Supplementary Methods) in 1% goat serum in TBS). Primary antibodies used were rabbit-anti-Transferrin-FITC (0.5 μg/mL; ab34670, Abcam, Cambridge, UK), mouse-anti-E-Cadherin (1:50; ab1416; Abcam), and rabbit-anti-CYP3A4 (1:500; AB1254; Merck-Millipore, Darmstadt, Germany). Secondary antibodies used were goat-anti-mouse (11029; Life Technologies; 1:1000) or goat-anti-rabbit Alexa Fluor 488 (11034; Life Technologies; 1:1000) combined with Hoechst 33342 (H21492; Life Technologies; 10 μg/mL) and Phalloidin-TRITC (R415; Life Technologies; 3 U/mL). Brightfield and fluorescent microphotographs were taken with an EVOS Auto FL imaging platform (Thermo Scientific), and subsequently processed with ImageJ 1.48 v (National Institute of Health, Bethesda, USA).

Immunostaining and confocal microscopy. For staining of the specific TJ-associated protein ZO-1, HepaRG cells were grown on coverslips in a 12 well plate and treated with APAP (as above). The cells were washed with PBS and then fixed by 10 minutes treatment with methanol (−20 °C). Fixed cells were washed in PBS containing 0.1% Tween 20 (PBS-T; Sigma-Aldrich). The cells were then blocked for 1 hour in 10% normal goat serum/0.1% PBS-T (Life Technologies. The cells were then washed twice before being treated with primary antibodies overnight at 4 °C. ZO-1 antibody (1:50; Santa Cruz; Insight Biotechnology, Middlesex, UK) was made up in 1% normal goat serum in 0.1% PBS-T. Following primary incubations, the cells were washed twice in PBS-T and then incubated with Alexafluor secondary antibody (Life Technologies) for 1 hour at room temperature. The secondary antibody was made up in PBS. Coverslips were washed twice in PBS-T, and twice in PBS, then mounted using Fluoroshield with DAPI (Sigma-Aldrich) onto slides. Cells were imaged using a Zeiss LSM700 Confocal Microscope (Carl Zeiss, Cambridge, UK) and Zen software.

Prestoblue live-cell viability and ATP-depletion endpoint hepatotoxicity assays. Following the 24 hour treatment with APAP, as described above, 10% (v/v) PrestoBlue® (A-13262; Life Technologies Paisley, UK) was added to cell culture medium and incubated for 30 minutes, before the fluorescence signal was measured on a GloMax-Multi + Microplate Multimode Reader (Promega Southampton, UK), and data processed, according to the manufacturer’s instructions. After performing the non-toxic PrestoBlue assay, remaining cells were lysed to determine total cellular ATP levels using the CellTiter-Glo® Luminescent Cell Viability Assay (G7570; Promega), as per vendor’s instructions. Bioluminescent signals were detected with the above plate Reader. ATP levels were normalized to controls.

### Markers of cellular stress and DILI in HepaRG cells following APAP: Lactate and Albumin production

Supernatant medium was collected after 24 h APAP (0–40 mM) and assayed for lactate or albumin^31^ as previously described^69^. Albumin synthesis was measured in cell culture supernatants after 24 h culture in serum-free William’s E Medium using an Albumin Blue 580 Fluorescence Assay as previously described^28^,^69^.

#### *Effects of phorbol ester on hepatic tight junctions*

Phorbol-12-myristate-13-acetate (PMA; P8139, Sigma-Aldrich) is known to disrupt integrity of hepatocellular TJs^4^. As a biochemical correlate with the impedance parameter, Rb (TJ barrier resistance); we tested effects of various concentrations (0–200 ng/ml) of the PKC-activator PMA using impedance monitoring for 24 hours on HepaRG cells.

#### *Flow Cytometry Analysis*

Integrin expression in response to APAP. To assess correlation of z-alpha with modulation of expression of integrins, adhesion molecules and other markers in response to APAP hepatotoxicity, remaining adherent HepaRG cells were recovered using TrypLE™ cell-dissociation reagent (Life Technologies), and stained using combinations of directly conjugated monoclonal antibodies: CD29-BV510 (563513), CD49f-BV421 (5625820, CD49d-FITC (580840), CD49c-PE, CD166-PE (559263) (all BD Biosciences, Oxford, UK); CD49a-APC-Vio770 (130-101-406), CD44-APC-Vio770 (130-099-149), CD49b-PE-Vio770 (130-100-328), CD90-PE-Vio770 (130-099-295), CD49e-APC (130-097-221), (all Miltenyi Biotec, Surrey, UK); CD13-BV421 (301716; Biolegend), CD54-FITC (mhcd5401; Caltag, Buckingham, UK). Cells were incubated with optimal concentration of antibodies (1/50) at 4 °C for 20 minutes, washed twice and resuspended in PBS containing 0.1% BSA and 0.1% sodium azide. Unstained cells were included as controls, and dead cells and debris were excluded from the analysis, based on scatter characteristics. Data for at least 10,000 live events per sample were acquired using a MACSQuant Analyzer (Miltenyi Biotec) and analyzed using FlowJo version 9.6.7 software (Flowjo LLC). Data is expressed as relative mean fluorescence intensity (MFI), and % positive staining.

### *In vivo* mouse model of APAP-induced liver injury

Animal work was approved by the University of Edinburgh College of Medicine & Veterinary Medicine Animal Ethics Committee and in accordance with the Animals (Scientific Procedures) Act 1986 and UK Home Office regulations (licence PPL 60/4320). After an overnight fast (12 hours), male mice (C57/BL6) aged 12–14 weeks, received acetaminophen by intra-peritoneal (IP) injection. Acetaminophen (Sigma) was dissolved in sterile phosphate-buffered saline (PBS) and warmed to 42 °C. Dosing range used was 75 mg/kg, 150 mg/kg and 300 mg/kg (at least n = 2 animals for each dose). Control animals received an equivalent volume of IP PBS. All animals were then allowed access to food and water *ad libitum*. Animals were harvested 24 hours after injection, at which point blood was taken by cardiac puncture for Liver Function Tests (LFTs) to analyse blood serum enzymes.

### Expression of ZO-1 following *in vivo* APAP treatment of mouse liver: Histopathology and immunohistochemical quantification

#### Cryotomy and immunohistochemistry

For immunohistochemistry (IHC), frozen samples were embedded in optimal cutting temperature compound (OCT) for cryosectioning, and sectioned at 5 μm thickness with a cryotome. This was followed by air drying at room temperature for an hour and fixation in acetone for 2 m at room temperature, PBS rinsing, and postfixation in periodate-lysine-paraformaldehyde (PLP) for 8 m at 4 °C. The sample was then rinsed serially in PBS, distilled water, and TBS before exposure to the primary antibody for 12 h at 4 °C at a 1/100 dilution (ZO-1 (H-300): sc-10804, Santa Cruz biotechnology, Inc., UK). This was followed by 20 m of exposure to Dako Real Blocker (Dako, UK). The envision kit was used for visualization, using 3,3′-diaminobenzidine (DAB) as the chromogen. All slides were counterstained with haematoxylin.

#### Microscopic examination and immunohistochemical quantification

All slides were evaluated by a boarded veterinary pathologist (JDP) and qualitative features of the staining recorded. In order to quantify the DAB stained percentage of each sample, three microscopic pictures were taken at x400 from an area adjacent to the central vein in randomly chosen lobules for each sample. In samples with centrilobular necrosis, this was not included in the picture, as cellular collapse resulted in concentration of positive staining (see results). A Fiji macro was developed for automatic DAB stain detection^70^. This macro included a background substraction rolling ball algorithm with a radius of 150pixels^71^, followed by colour deconvolution calibrated to DAB and haematoxylin stains in single stained slides^72^, thresholding (0–170), and area measurement. Before final data collection, the macro was refined and validated in a randomly chosen subset of pictures for each experimental group. Comparison of different parameter combinations was achieved by overlaying the measured area to the original picture^73^. The amount of positive staining is expressed as the percentage area of positive stain relative to the total area of the picture (*i.e.* 225123 μm^2^). Differences between groups were assessed with Kruskal-Wallis, with a significance threshold of p < 0.05.

Morphological and ultrastructural assessment. To examine ultrastructural features of APAP toxicity using transmission electron microscopy (TEM), cells were cultured in 6-well plate format and treated for 24 hours (as above), then processed as previously described^28^.

#### Statistical analysis and data presentation

All data are represented as mean ± standard error. One-way Anova and Tukey-Kramer multicomparison tests were used to determine whether the groups under investigation were significantly different from each other. Probability levels of P < 0.05 and P < 0.01 were set as significant. For immunohistochemical quantification, differences between groups were assessed with Kruskal-Wallis, with a significance threshold of P < 0.05. Impedance spectral modeling data analysis: Impedance data were analysed using Matlab (see Supplementary Information).

## Additional Information

**How to cite this article**: Gamal, W. *et al*. Low-dose acetaminophen induces early disruption of cell-cell tight junctions in human hepatic cells and mouse liver. *Sci. Rep.* **7**, 37541; doi: 10.1038/srep37541 (2017).

**Publisher's note:** Springer Nature remains neutral with regard to jurisdictional claims in published maps and institutional affiliations.

## Supplementary Material

Supporting Information
