# Supplementary material for: Low-dose acetaminophen induces early disruption of cell-cell tight junctions in human hepatic cells and mouse liver
Source: Sci Rep. 2017 Jan 30;7:37541. doi: 10.1038/srep37541 (PMC5278402; doi:10.1038/srep37541)
Supplement: Supporting Information [file srep37541-s1.pdf]

Supporting Information for

Low-dose acetaminophen induces early disruption of cell-cell tight junctions in human hepatocytes and mouse liver

*Wesam Gamal[1] and *Philipp Treskes[2], Kay Samuel[3], Gareth Sullivan[4,5,6], Richard Siller[4], Vlastimil Srsen[1], Katie Morgan[2], Anna Bryans[2], Ada Kozlowska[2], Andreas Koulovasilopoulos[2], Ian Underwood[7], Stewart Smith[1], Jorge del-Pozo[9], Sharon Moss[9], Alexandra Inés Thompson[10], Neil C Henderson[10], Peter Clive Hayes[2], John N Plevris[2], †Pierre-Olivier Bagnaninchi[1] and †ξLeonard Joseph Nelson[2]

[1]MRC Centre for Regenerative Medicine, SCRM Building, The University of Edinburgh, Edinburgh BioQuarter, 5 Little France Drive, Edinburgh EH16 4UU; [2]Hepatology Laboratory, University of Edinburgh, Royal Infirmary of Edinburgh, 49 Little France Crescent EH16 4SB; [3]Scottish National Blood Transfusion Service, Research, Development and Innovation Directorate, Cell Therapy Group, Ellens Glen Road, Edinburgh, EH17 7QT; [4]Department of Biochemistry, Institute of Basic Medical Sciences, Faculty of Medicine, University of Oslo, PO Box 1112 Blindern, 0317 Oslo, Norway; [5]Norwegian Center for Stem Cell Research, PO Box 1112 Blindern, 0317 Oslo, Norway; [6]Institute of Immunology, Oslo University Hospital-Rikshospitalet, PO Box 4950 Nydalen, Oslo 0424, Norway; [7]Institute for Integrated Micro and Nano systems, University of Edinburgh, Scottish Micro Electronic Centre, Alexander Crum Brown Road, EH9 3FF; [8]Institute for Bioengineering, University of Edinburgh, King's Buildings, Colin MacLaurin Road, EH9 3DW; [9]Easter Bush Pathology, The Royal (Dick) School of Veterinary Studies and The Roslin Institute, Easter Bush Campus, Midlothian, EH25 9RG; [10]MRC Centre for Inflammation Research, The Queen's Medical Research Institute, University of Edinburgh, Edinburgh EH16 4TJ, UK.

Supplementary Methods

Methods overview

Electric Cell-substrate Impedance Sensing

We utilized the commercial system Electric Cell-substrate Impedance Sensing (ECIS-Zθ, Applied Biophysics, Troy, NY USA), first to characterize establishment of the organotypic (HepaRG co-culture [1] liver model on the microelectrodes, and then to study dose-/ time-dependent effects (0-24 hours) of APAP on cellular parameters of TJ, cell-substrate adhesion, and membrane integrity. A technical workflow, outlining the HepaRG-based model, and subsequent impedance spectral data deconvolution, into biologically-relevant parameters of cell behaviour, are shown in **Fig. 2 and Supplementary Figure 1**. Parallel assessment of cellular morphology, phenotype, gene and protein expression, alone, or in the context of APAP hepatotoxicity in HepaRG cells, were used to provide correlation with impedance-based quantitative measurements, and liver biochip system validation. Furthermore, effects of both phorbol ester (PKC-activator which abrogates hepatic TJs integrity) and purified NAPQI, on HepaRG TJs were investigated. See Supporting Information (below) for methodological details of: HepaRG and PHHs cell culture on ECIS microelectrodes; hepatotoxicity assays; flow cytometry analysis; immunocytochemistry; morphological and ultrastructural assessment; and impedance spectral modeling data analysis.

Impedance-based cellular assays (IBCAs) represent an emerging technology in drug discovery, due to their high sensitivity and quantitative nature. Pioneered by Giaever and Keese [2], impedance sensing can detect minute changes in the behaviour of cells directly cultured on micro-electrode arrays. Impedance sensing is utilized mostly as a global indicator of cellular status, called Cell Index, and has been applied across many fields of biology such as, cell-substrate attachment and spreading [3,4], signal transduction [5] , cytotoxicity [6] , metastasis [7], cardiology [8], and regenerative medicine [9]. Very rarely however, is impedance-sensing fully exploited in its spectroscopic form, to retrieve biologically meaningful electrical parameters, such as paracellular and transcellular resistance. Typically, IBCAs have found a niche application in blood-brain barrier permeability studies, where impedance sensing has provided real-time quantitative monitoring of the establishment or loss of cell-cell endothelial TJs [3].

Impedance spectral modeling data analysis

Impedance data were analysed using Matlab. For clarity, to analyze differences in impedance curves (**Fig. 2, Fig. 3,** and **Fig. 4**), we used *normalized* values, calculated as impedance values from the microelectrode confluent with HepaRG cells, divided by its value at the challenge starting point (t=0). For comparison, absolute impedance values (termed, '*non-normalized'* data) for all parameters (Global, Z' (**Ω**); Rb (**Ω.cm^2**); z-alpha (**Ω$^{0.5}$cm)**; and Cm (**µF/cm^2**)), are provided, with statistical significance, for APAP (**Supplementary Table 1 and Supplementary Table 3)**; and for PMA **(Supplementary Table S3 and Supplementary Fig. 4)**.

Culture of Primary Human Hepatocytes on ECIS microelectrodes and treatment with acetaminophen following rifampicin induction

ECIS 8W10E+ arrays were pre-coated with collagen, as per manufacturer's instructions (Biopredic Int.), before seeding with primary human hepatocytes (PHH). PHH were seeded at 200, 000 cells per well and incubated at 37°C for 24h to allow for cell attachment. Plates were then connected to ECIS and placed in the incubator to quantitatively monitor PHH growth

kinetics using impedance measurements. Cells were exposed to Rifampicin, a known CYP3A4 inducer, for 24h prior to a 24h APAP hepatotoxicity assay. The effect of 0, 5, 10 and 20mM APAP on PHH impedance measurements was then monitored for 24h. ECIS modelling was then performed to deconvolve the impedance into its different parameters, in order to ascertain the effect of APAP on tight junctions, cell adhesion and cell membrane integrity.

Hepatotoxicity assays: primary human hepatocytes (PHHs)

A parallel study was conducted using a collagen-coated 96 well plate (Corning™ BioCoat™ Collagen I) to correlate ATP (industry standard endpoint) and PrestoBlue (live-cell) assays, with the ECIS measurements. PHHs were seeded at 50,000 cells per well. Cells were induced using Rifampicin for 24 hours before APAP hepatotoxicity was tested at 0, 5, 10 and 20mM as described above. The plate was removed from the incubator to equilibrate to room temperature (20°C). PrestoBlue solution was prepared in media using a 9:1 dilution and 100µl was added to each well in addition to 4 no-cell control wells. The plate was then incubated at room temperature for 30 minutes in the dark in order to preserve the fluorescent signal. After 30 minutes, the plate was read using GloMax: Protocol PrestoBlue. The plate was washed with 100µl media per well and 4 no-cell control wells were selected before an ATP assay was performed according to manufacturer's instructions (Promega. CellTitreGlo).

Supplementary Figures 1-10

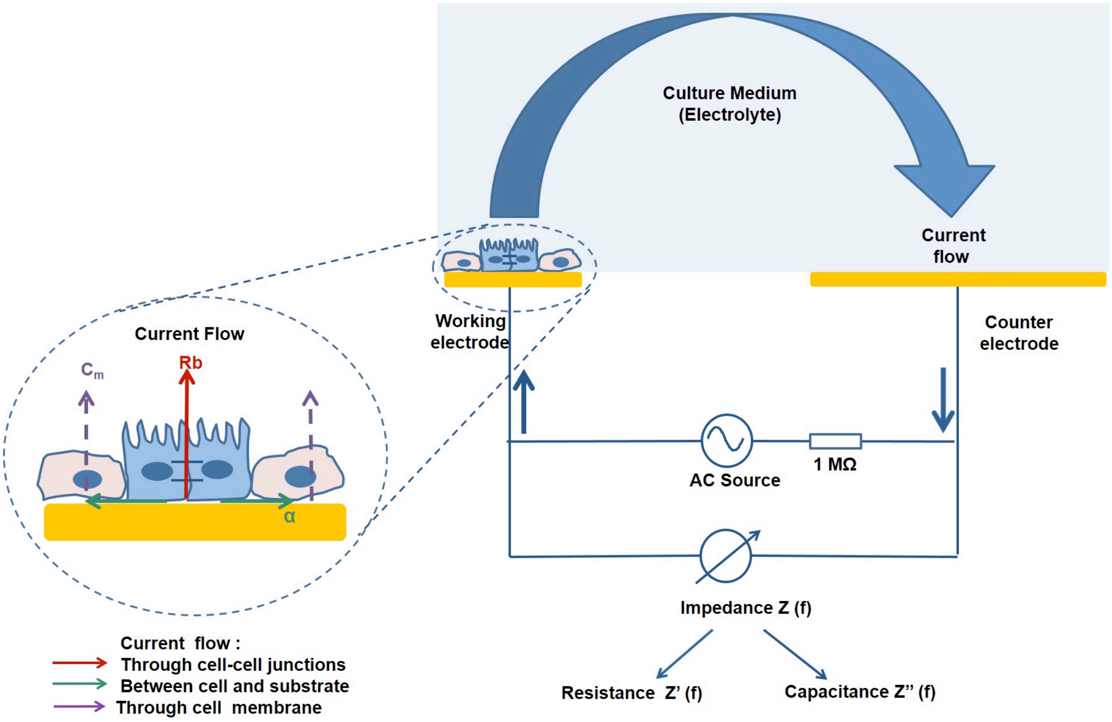

Supplementary Figure 1 Electric Cell-substrate Impedance Sensing Model

Schematic showing basic principles underlying ECIS measurements: Electric Cell-substrate Impedance Sensing (ECIS) is a method used to monitor and characterise cells cultured on top of microelectrodes. The impedance of the cells is measured by driving a small alternating current (AC) signal between the sensing (working) electrode, and a larger counter electrode. A lock-in amplifier is then used to measure the variations in the complex impedance, composed of real (resistive) and imaginary (capacitive) parts. As cells grow on top of the sensing electrode, they impede current flow resulting in an increase in the measured complex impedance. Hence, any change in cell morphology or behaviour is mirrored by the impedance measurements. With reference to **Supplementary Fig. 1** outlining impedance spectral modeling (see **Fig**, **2** for comparison**)**, the ECIS-Zθ system scans impedance measurements through various frequencies to recognize the current pathways, and translates spectral data (Z') by deconvolution, using the built-in mathematical model (ECIS-Zθ software), into biologically-relevant *cell electrical parameters*: **Rb** (cell-cell junctions), **z-alpha** (cell-electrode adhesion) and **Cm** (cell membrane capacitance). This is based on the fact that at low frequencies, the current flows underneath and in-between the cells, reflecting both relative tightness of cell-cell junctions, and the degree of cell adherence to the electrodes. At high frequencies, the current can capacitively couple through the cells, revealing data on intracellular properties and integrity of the cell plasma membrane.

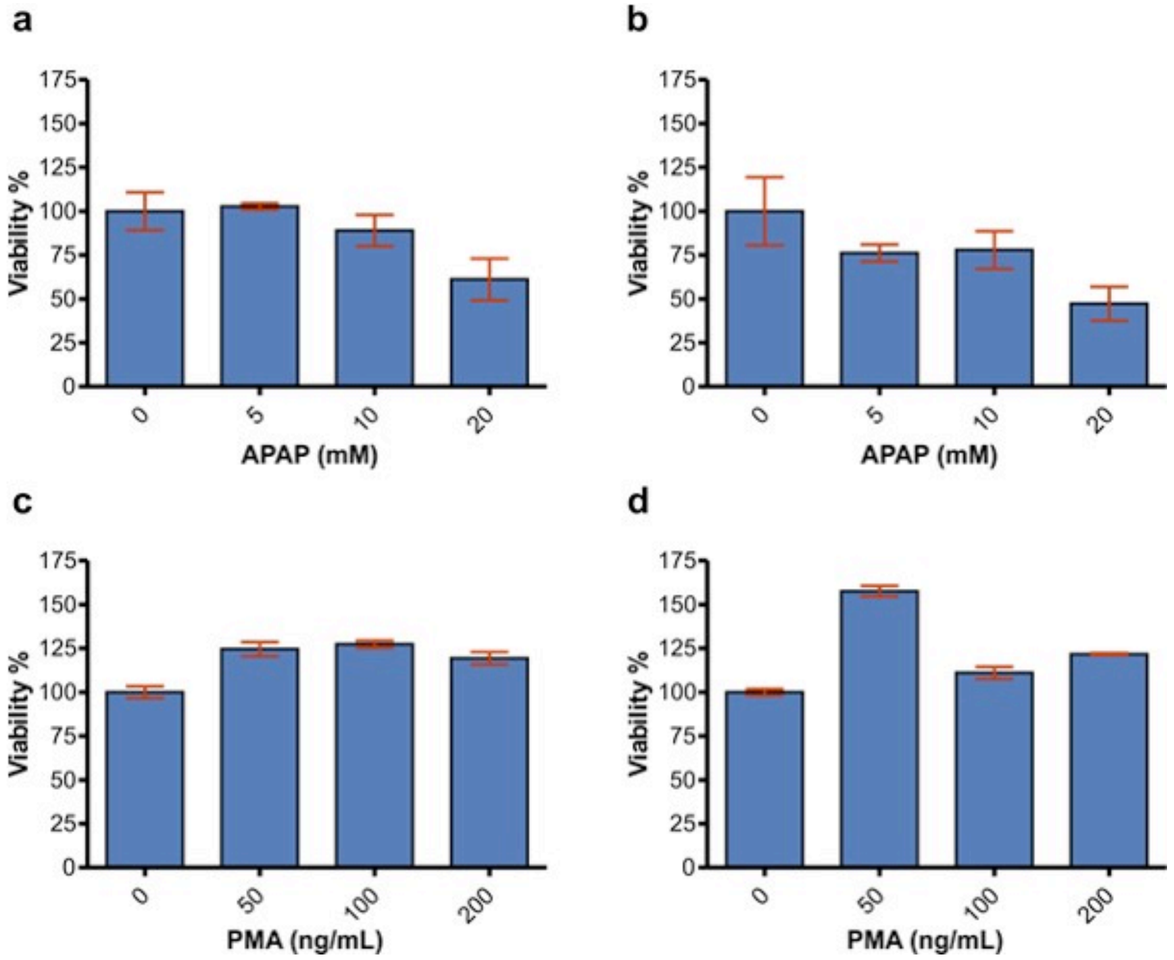

Supplementary Figure 2 Hepatotoxicity assays following 24 hours APAP or PMA treatment

To assess hepatotoxic response of APAP or PMA in HepaRG cells and for comparison with impedance measurements (see **Fig. 3, Fig. 4**), dose-dependent hepatotoxicity was detected at 24 hours, by multiplexing ATP-depletion endpoint assay (**a**, **c**) with the PrestoBlue live-cell viability assay (**b**, **d**). Prestoblue and ATP values are expressed relative to the levels found in control cells, arbitrarily set at a value of 100%. ATP content decreased to only 50% of control values at 20 mM APAP (**a**); with minimal change observed at 5-10 mM. Cell metabolic activity, as measured by Prestoblue reduction (providing also a quantitative measure of viability and cytotoxicity), was more pronounced in sub-toxic (5 mM) and intermediate (10 mM) dose APAP, compared with ATP-depletion assay. PMA caused moderate increases of both ATP-content (**c**), and PrestoBlue live-cell viability (**d**), at 24 hours; possibly due to hormetic effects of the PKC activator, PMA.

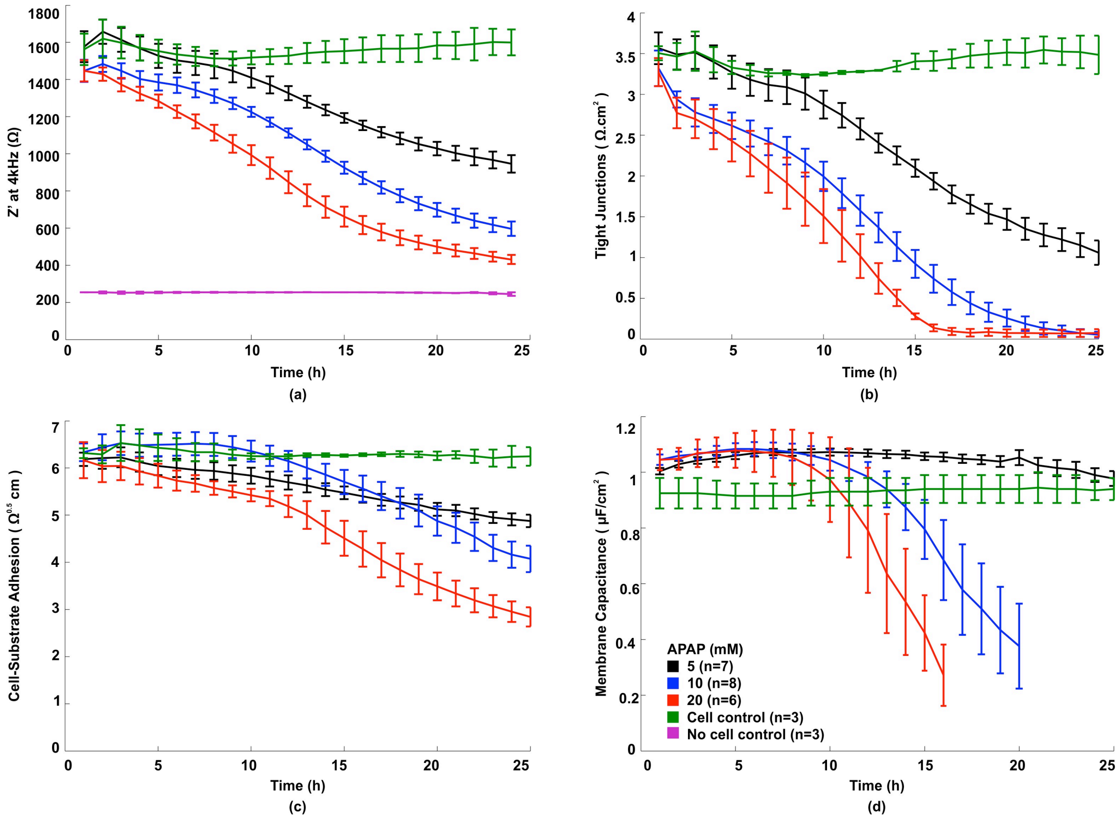

Supplementary Figure 3 Real-time impedance monitoring (0-24 hours) of HepaRG-based liver-on-chip device following 24 hours APAP challenge: Non-normalized dose response

Effect of APAP on non-normalized impedance data in real-time. Comparative impedance spectral modeling data is plotted; expressed as absolute impedance values (termed, '*non-normalized'* data) for all parameters (Global, Z' (**Ω**); Rb (**Ω.cm^{2}**); z-alpha **(Ω$^{0.5}$cm)**; and Cm **(µF/cm2)**. Absolute values, with statistics are shown in **Supplementary Table 1**. The dose- and time-dependent decline in the overall resistance is shown in (**a**) while the effect of APAP on tight junctions, cell-substrate adhesion and cell membrane capacitance is illustrated in (**b**), (**c**) and (**d**), respectively. Both the non-normalized and normalized data (**Fig. 3**) showed a concentration- and time-dependent decrease in the resistance (Z') and the modeling parameters (Rb; z-alpha; Cm). Subsequently, data was normalized through dividing the impedance (or its deconvolved parameters) by its value at the APAP challenge starting point (t=0); see corresponding normalized data in **Fig. 3**.

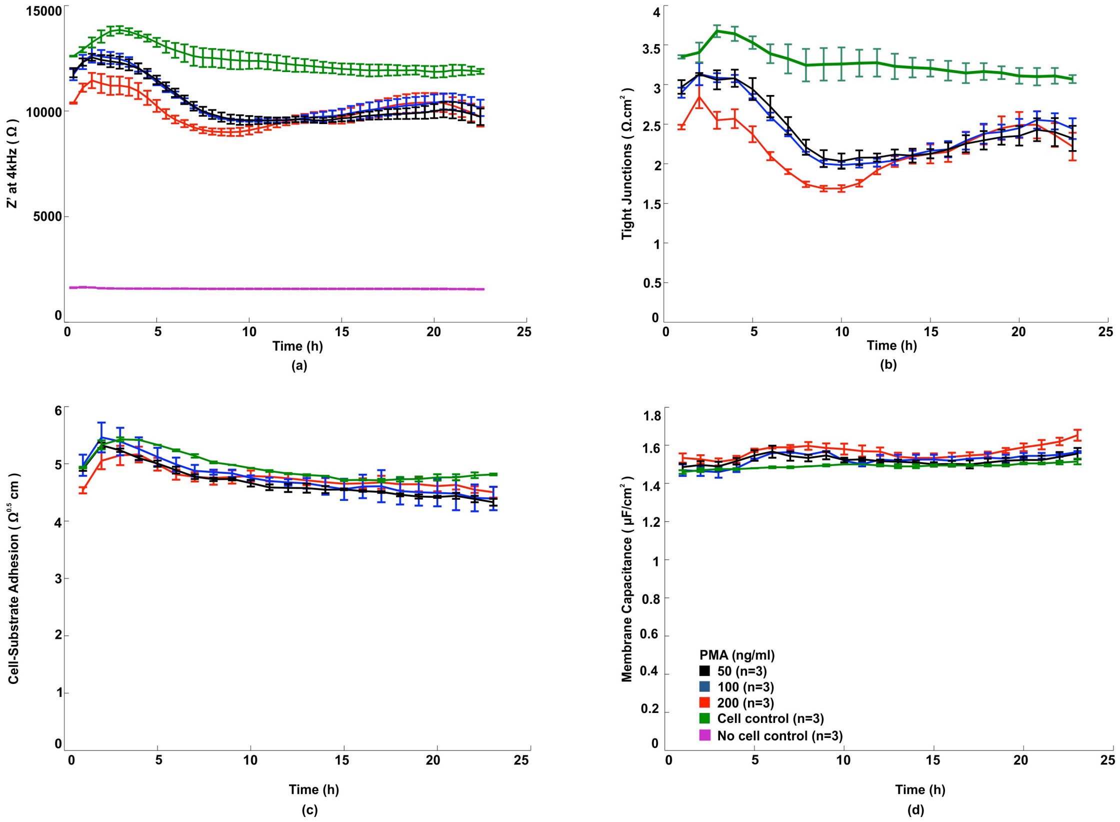

Supplementary Figure 4 Real-time impedance monitoring (0-24 hours) of HepaRG-based liver-on-chip device following 24 hours phorbol-ester challenge: Non-normalized dose response

a-c The spike is characteristic of a change of media and a peak in impedance (due partly to a change in pH, temperature, and dissolved CO2; as observed in no cell control), but predominantly to the ensuing cell response; possibly due to hormesis. **(a)** Non-normalized resistance initially showed an increase to phorbol-ester, followed by significant decreases (by 12 hours); then an increase back to the original pre-challenge values. **(b)** The most substantial effect of phorbol-ester was on Rb (cell-cell tight junctions), which decreased at all doses tested (3-8 hours); and showed significant decreases (≤6 hours) at 100 ng/ml ($P<0.05$) and 200ng/ml ($P<0.01$); (**Supplementary Table 3)**, returning close to original values. **(c-d)** No significant effects of phorbol-ester were detected on either cell-electrode adhesion (z-alpha), or Cm (cell membrane integrity). Absolute values, with statistics are shown in **Supplementary Table 3**. See corresponding normalized data in **Fig. 4**.

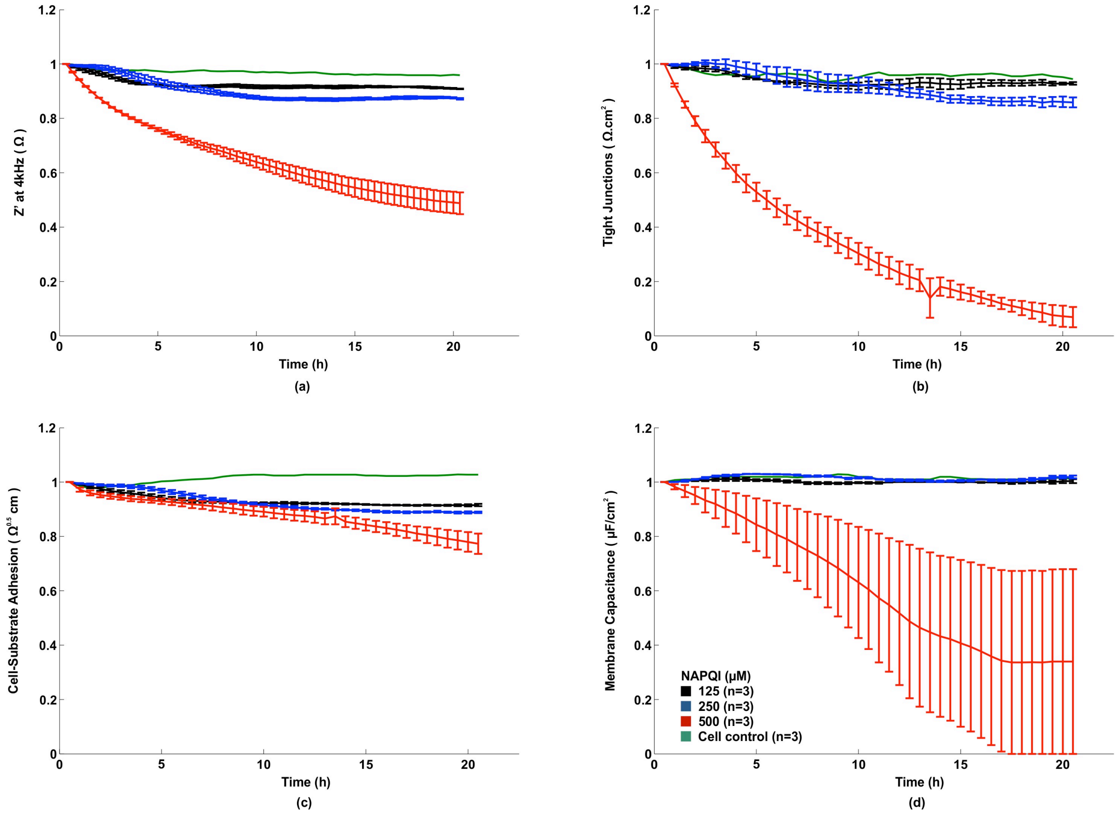

Supplementary Figure 5 Real-time impedance biosensing (0-20 hours) data in HepaRG cells following treatment with NAPQI (0, 125, 250, 500 µM) for 20 hours

NAPQI formation is the major cause of APAP hepatotoxicity, we therefore tested direct effects of different concentrations of NAPQI on TJs (Rb parameter) with impedance sensing. At higher dose NAPQI (500 µM), non-normalized resistance showed an approximate 50% reduction over 20 hours (**a**), indicating a decline in cellular health. An abrupt (0-2 hours) and sustained (0-20 hours) disruption of TJs occurred (**b**), with a 50% decrease in the impedance parameter (Rb) after 6 hours. Z-alpha also declined by 20% after 20 hours (**c**). Minimal effects recorded on both Rb and z-alpha (at 125 and 250 µM), may reflect detoxification of NAPQI by intrinsic stores of glutathione (GSH), as high doses of APAP are required to deplete mitochondrial GSH. The apparent abberrant effect on Cm at high dose NAPQI, could be due to the labile nature of NAPQI, formation (over hours) of protein adducts, causing lipid peroxidation of the cell membrane.

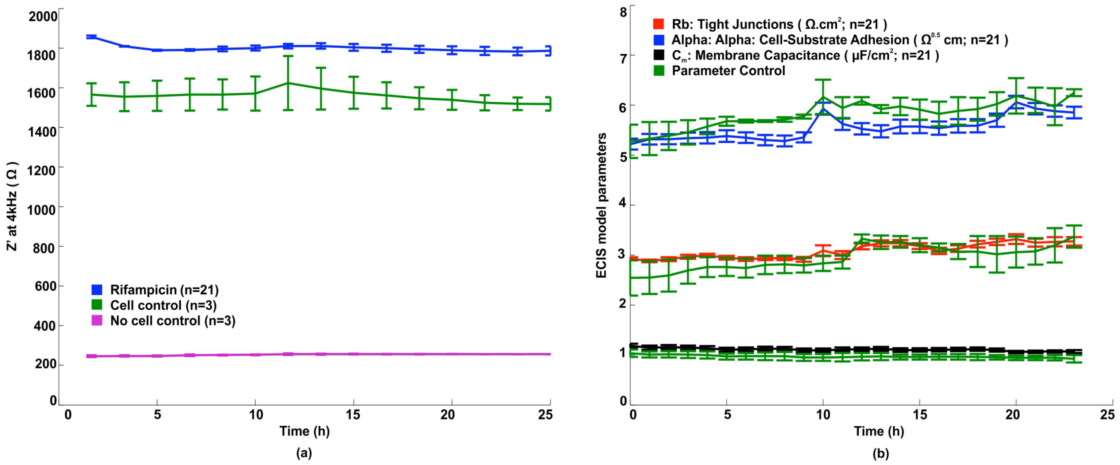

Supplementary Figure 6: Impedance measurements in HepaRG cells following 24 hour rifampicin

All APAP toxicity assays were preceded by an induction phase of rifampicin (CYP3A4 inducer). Rifampicin showed minimal effect on HepaRG cells as monitored by impedance biosensing. (**a**) Effect of rifampicin on resistance: Rifampicin induction did not have any deleterious effects on global cell health, as reflected by impedance measurements. (**b**) Similarly, Rifampicin induction did not cause any disruption to impedance-modeled parameters, Rb (cell-cell tight junctions), z-alpha (cell-substrate adhesion) or Cm (cell membrane integrity).

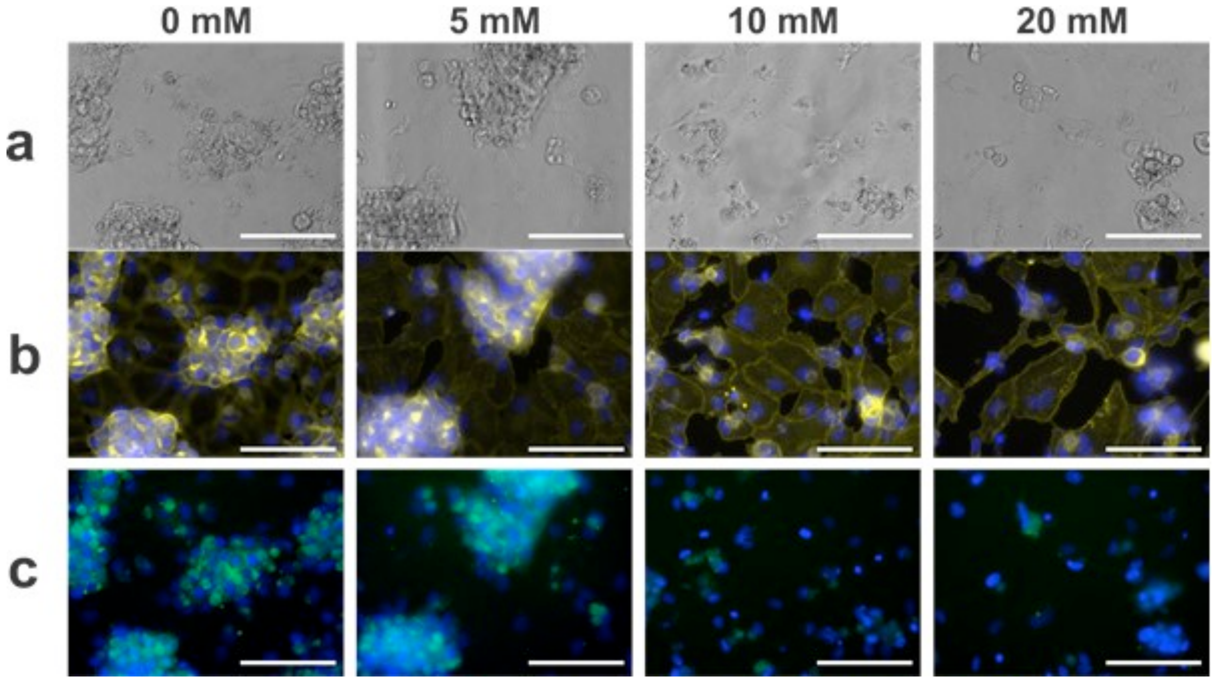

Supplementary Figure 7 Disruption of hepatic architecture in HepaRG cells following 24 hours APAP treatment: Co-localization F-actin-phalloidin/ E-cadherin immunofluorescence staining

Phase-contrast images of HepaRG hepatocyte (H)/ cholangiocyte (Ch) co-culture (**a**), and corresponding immunofluorescent structural cytoskeletal F-actin (**b**), and E-caherin staining, (**c**). (**a**) Characteristic *in vivo*-like hepatic cord (H) phenotype in control cultures (0 mM) is progressively lost with increasing APAP concentration. (**b**) Strong F-actin/ Phalloidin staining (yellow; Hoechst nuclear counter-stain, blue) reveals distinct pericanalicular-cytoskeletal F-actin bands (arrows) in control cells and sub-toxic (5 mM) APAP; with less intense staining around the canalicular region at higher APAP dose; consistent with a direct effect of APAP reactive metabolites on actin structures. (**c**) Co-localization staining of E-cadherin (green – overlayed with Hoechst nuclear stain in blue), a junctional-associated protein and marker of lateral polarized phenotype, confirms gradual disruption of gross hepatic culture morphology with increasing APAP.

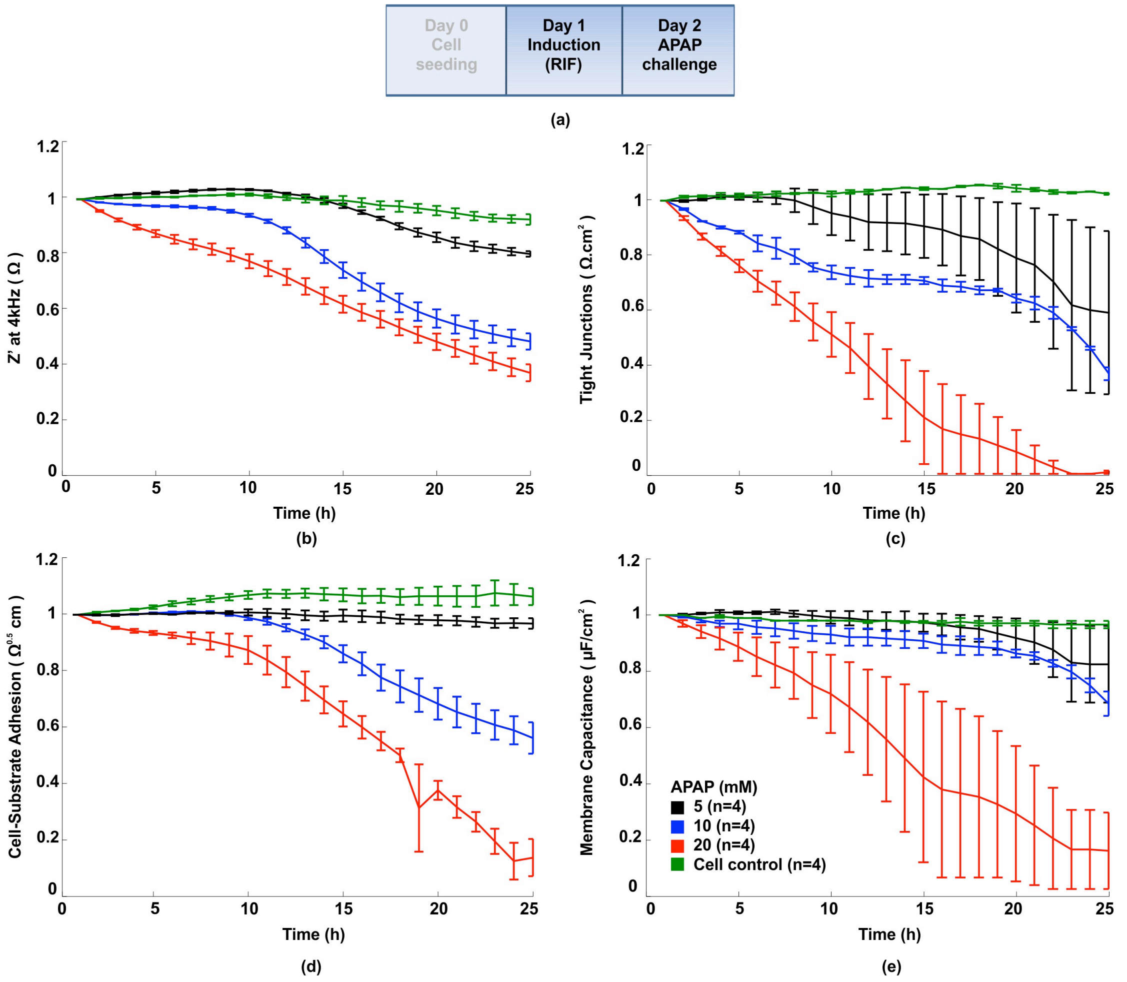

Supplementary Figure 8 Real-time impedance monitoring of primary human hepatocytes cultured on ECIS arrays following 24 hour APAP challenge

(a) Protocol time line: Following 24 hours rifampicin induction in confluent primary human hepatocytes (PHHs), cells were treated for 24 hours with APAP. (inset key: untreated cell control; 5 mM; 10 mM; and 20 mM). (**b**) Post-challenge resistance kinetics: APAP caused a dose-dependent decline in normalized resistance - a global indicator of cellular status. **(c) Rb (cell-cell tight junctions)**: APAP disrupted TJs in a concentration- and time-dependent manner; compared with control values, Rb decreased sharply (by 6 hours) at 10 and 20 mM APAP. (**d**) **z-alpha: Cell-substrate adhesion** disruption was detected early at intermediate (10 mM) and high dose (20 mM) APAP, suggesting loosening of cells from the electrode surface. (**e**) Quantitative **Cm** values (membrane capacitance), reflecting cell membrane integrity, were significantly compromised both at high APAP doses (10-20 mM), and earlier (12 hours), than those of cell viability measured in corresponding 24 hour biochemical toxicity assays. See **Supplementary Table 1** and **Supplementary Figure 3** for corresponding non-normalized data.

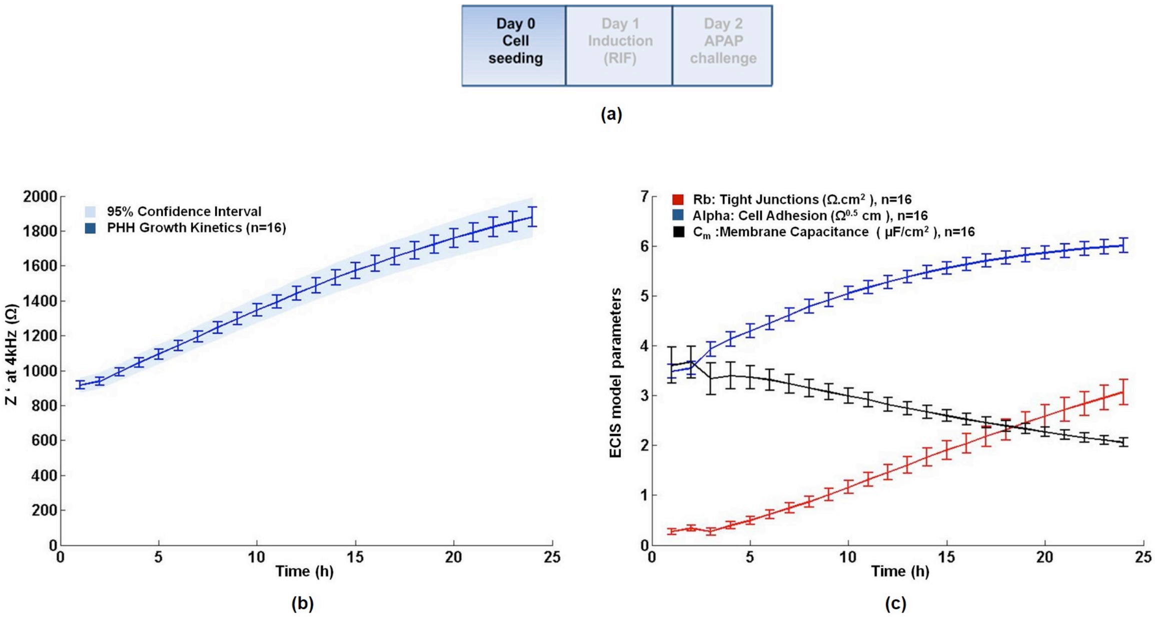

Supplementary Figure 9 Real-time quantitative impedance monitoring of primary human hepatocyte growth kinetics

(**a**) Time-line protocol: cells were seeded on Day 0 with a density of 200,000 primary human hepatocytes (PHHs)/cm^2. Cells were induced with rifampicin on Day 1 (24h post-seeding) for 24h before conducting the 24h APAP challenge at the concentrations indicated. (**b**) Non-normalized impedance showing an increase in impedance commensurate with cell growth, maturation and establishment of cell adhesion structures (Rb and z-alpha). (**b**) The increase in impedance with PHH growth can be attributed to the formation of cell tight-junctions and cell adhesion indicated by the increase in the modelled R_b and z-alpha. The slight decrease in cell-membrane capacitance C_m might be due to the fact that, upon isolation, PHHs are in a state of 'pre-apoptotic cell stress' – before recovery in culture [10] .

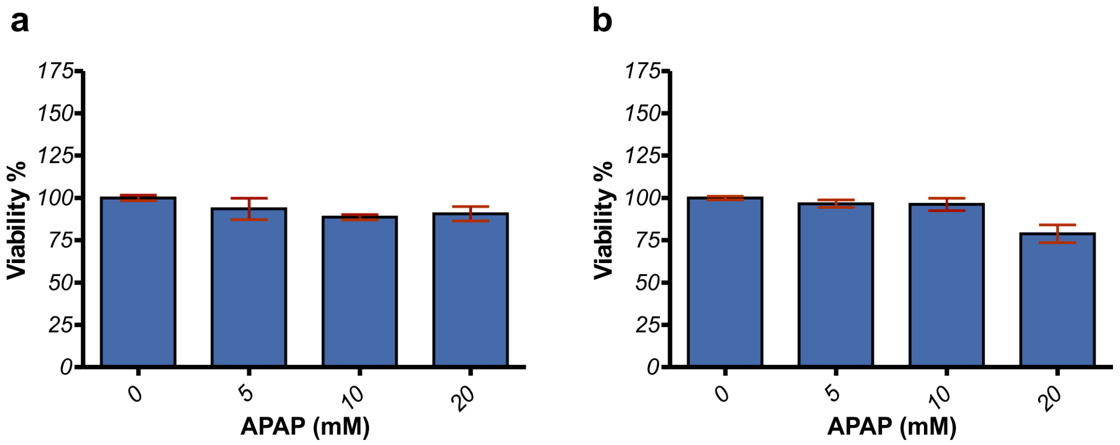

Supplementary Figure 10 Primary human hepatocytes hepatotoxicity assays following 24h APAP Challenge.

Minimal changes were observed in cell viability of primary human hepatocytes using either industry standard endpoint (ATP; panel **a**) or live-cell (Prestoblue; Panel **b**) hepatotoxicity assays, even at 20mM APAP.

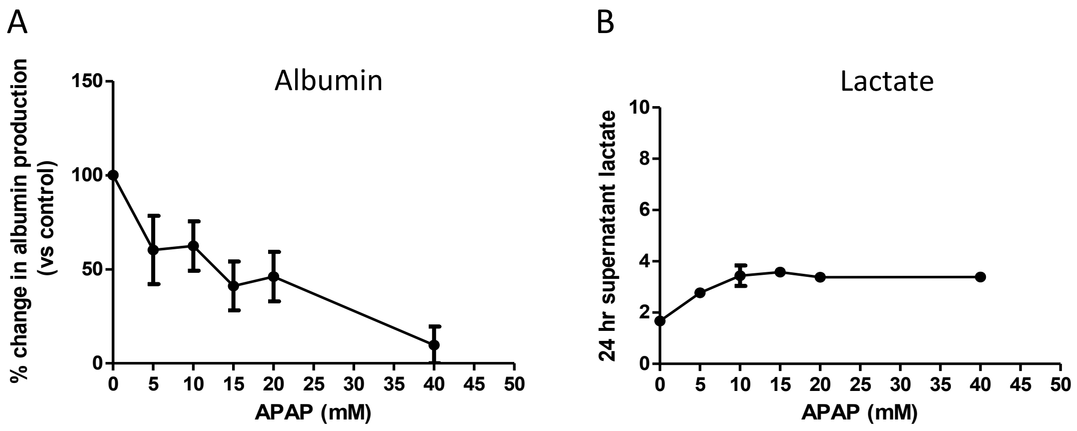

Supplementary Figure 11 Hepatotoxicity assays for albumin and lactate production following 24 hours APAP treatment in HepaRG cells

To assess hepatotoxic response of APAP (0-40 mM) in HepaRG cells and for comparison with impedance measurements (see **Fig. 3**), dose-dependent hepatotoxicity was detected at 24 hours, by assessing albumin (**A**) and lactate (**B**) levels, as previously described[11,12]. Values for albumin shown are expressed relative to the levels found in control cells, arbitrarily set at a value of 100%. Values for lactate are in mmol/ L. In HepaRG cultures, albumin production decreased from 0.36 (untreated) to 0.04 (40 mM) ug/h/10^6 viable cells seeded. Conversely, lactate release increased sharply at 5mM APAP, and according to dose (5-15 mM), and plateaued at 20-40 mM.

Supplementary Tables 1-3

APAP Challenge	Control	6 hrs	12 hrs	24 hrs
**$Z'(\Omega)$**				
5 mM	1680.90 ± 126.82	1603.00 ± 73.60	1346.10 ± 34.30*	930.92 ± 95.27**
10 mM	1508.30 ± 74.02	1418.50 ± 49.13	1042.28 ±36.60**	585.45 ± 72.24**
20 mM	1513.80 ± 101.30	1191.20 ±52.68**	706.70 ± 60.10**	421.60 ± 20.40**
**R_b (Ω.cm)**				
5 mM	3.58 ± 0.36	3.53 ± 0.25	2.60 ± 0.10**	1.13 ± 0.32**
10 mM	3.25 ± 0.33	2.37 ± 0.28**	1.15 ± 0.26**	0.18 ± 0.08**
20 mM	3.45 ± 0.23	1.78 ± 0.30**	0.20 ± 0.09**	0**
**$\alpha(\Omega^{0.5}cm)$**				
5 mM	6.40 ± 0.20	6.30 ± 0.16	5.88 ± 0.08	2.97 ± 0.29**
10 mM	6.32 ± 0.24	6.75 ± 0.30	5.84 ± 0.08	2.67 ± 0.67**
20 mM	6.27 ± 0.69	5.93 ± 0.10	4.69 ± 0.37	1.49 ± 0.75**
**$C_m(\mu F/cm^2)$**				
5 mM	1.01 ± 0.01	1.06 ± 0.01	1.06 ± 0.02	1.04 ± 0.02
10 mM	1.03 ± 0.04	1.08 ± 0.05	0.9 0± 0.10	0**
20 mM	1.10 ± 0.07	1.06 ± 0.14	0.36 ± 0.22*	0**

Significantly different from control: * P<0.05; ** P<0.01

Supplementary Table 1: Real-time impedance monitoring (0-24 hours) of HepaRG-based liver-on-chip device following 24 hours APAP challenge: **Non-normalized impedance data** Table showing effect of APAP on non-normalized impedance values (Z', Rb, alpha and Cm), at different time points (6, 12, 24 hours). At 20 mM APAP, the decline in resistance (Z') is highly significant even after only 6 hours (P<0.01), indicating reduction in global cellular health. After 12 hours, the decrease in Z' is significant for the other doses (P<0.01) and at the end of the 24 hours assay, the resistances reach their minimum values, close to that of the cell free electrode for high (10-20 mM) doses (~300 Ω). The main effect of APAP is on the TJ parameter, Rb. At 6 h, highly significant decreases in Rb were measured for high dose (10-20 mM) APAP (P<0.01). At 12h, the decrease in Rb is significant even for the 5 mM dose with complete abolition of TJs at 10 and 20 mM APAP. Significant effects of APAP on cell-substrate adhesion (z-alpha), occurs later (>12 hours) than the effect on cell-cell TJs. At 6h, no significant decrease in adhesion is detected, while at 12h the 20 mM dose shows a significant decrease, and at 24 hours both the high doses (but not the low 5 mM dose), show a significant decrease in cell-substrate adhesion. The effect of APAP on the integrity of the cell membrane is only monitored for the high doses. It is more pronounced at 24 hours where Cm reaches zero, as membrane integrity is completely compromised, presumably with cell death and detachment. See corresponding **Fig. 3**.

Cell surface markers	APAP (mM)							
	0		5		10		20	
	%	MFI	%	MFI	%	MFI	%	MFI
**Integrins**								
CD29 high	60.3	8450	42.6	10037	59.6	9495	30.9	10398
CD29 low	39.7	2915	57.4	645	40.4	1668	69.1	1891
CD49a high	93.1	13780	75.4	12891	88.2	13013	92.9	11389
CD49a low	6.4	1530	23.2	647	10.8	989	6.8	722
CD49b high	94.3	16668	75.0	11278	85.8	13347	86.3	11866
CD49b low	4.2	848	16.6	645	10.7	776	9.9	776
CD49c high	43.0	20901	49.4	40328	33.9	32681	27.8	55263
CD49c low	57.0	2098	59.3	2187	66.1	2279	72.2	3196
CD49d	0.7	131	0.5	91	0.8	64	0.5	102
CD49e	93.8	12322	84.0	6984	86.4	8624	94.1	8650
CD49f	26.3	2888	10.6	2944	25.3	2235	4.2	3499
**Other markers**								
CD13 high	96.2	50902	61.3	70747	39.5	73510	28.9	86849
CD13 low	3.8	947	38.7	3717	60.5	6880	71.1	7826
CD44 high	55.2	66736	52.0	59345	32.6	73835	29.9	70751
CD44 int					25.7	8328	23.6	9135
CD44 low	41.5	2378	40.1	1157	30.7	347	33.0	361
CD54 high	71.2	10543	75.7	5573	58.3	4923	37.7	6590
CD54 low	28.8	2002	24.3	290	41.7	165	62.3	482
CD90 high	68.2	91722	54.4	98271	43.8	98783	30.8	55688
CD90 low	18.6	3554	32.3	4918	45.3	8675	53.6	3621
CD166 high	89.7	4437	58.3	7338	51.2	7160	31.5	10115
CD166 low	12.6	508	41.7	531	48.8	728	68.5	1258

Supplementary Table 2 Flow cytometry data of cell surface expression marker panel in HepaRG cells following APAP treatment (0, 5, 10, 20 mM) for 24 hours Data is expressed as relative mean fluorescence intensity (MFI), and % positive staining. Data for integrin expression panel (CD29; CD49a-f) is shown. Untreated HepaRG cells stained for expression of CD90, CD13, CD54, CD166, CD54 and CD44, showed low and high expressing sub-populations. Increasing APAP concentration was associated with increased percentage of low expressing sub-population. For CD44 the percentage of high and low expressing populations after treatment with 5 mM APAP was comparable to untreated control. However, treatment with 10 and 20 mM APAP decreased the percentage of cells in both low and high expressing populations resulting in the appearance of a population with intermediate expression. The MFI for cells with low expression of CD44 showed little change following APAP treatment, but decreased in the CD44 high population, suggesting that cells in this population, at 10 and 20 mM APAP, down regulated expression, emerging as the CD44 intermediate population. See corresponding histograms **Fig. 5**.

PMA Challenge	Control	6 hrs	12 hrs	24 hrs
**Z'(Ω)**				
50 ng/ml	8415.60 ± 137.35	7779.90± 143.90	6943.50± 116.60**	7447.10 ±260.07*
100 ng/ml	8339.20 ± 151.10	7582.40 ± 296.50	6846.20 ± 94.17**	7666.50 ± 218.50
200 ng/ml	7546.90 ± 58.38	6803.70 ±44.35**	6459.80 ± 146.23**	7509.10 ± 308.90
**R_b (Ω.cm)**				
50 ng/ml	2.97 ± 0.08	2.62 ± 0.09	2.09 ± 0.07**	2.43 ± 0.14*
100 ng/ml	2.90± 0.07	2.52 ± 0.02*	2.01 ± 0.06**	2.55 ± 0.10*
200 ng/ml	2.46 ± 0.03	2.01 ± 0.01**	1.77 ± 0.06**	2.49 ± 0.17
**α($\Omega^{0.5}$cm)**				
50 ng/ml	4.80 ± 0.02	4.84 ± 0.08	4.61 ± 0.05	4.50 ± 0.05
100 ng/ml	4.93 ± 0.11	4.92 ± 0.08	4.66 ± 0.04	4.56 ± 0.18
200 ng/ml	4.53 ± 0.05	4.79 ± 0.08	4.79 ± 0.07	4.63 ± 0.08
**C_m(μF/cm^2)**				
50 ng/ml	1.48 ± 0.02	1.56 ± 0.03	1.52 ± 0.01	1.52 ± 0.02
100 ng/ml	1.47 ± 0.02	1.56 ± 0.01	1.51 ± 0.01	1.53 ± 0.02
200 ng/ml	1.53 ± 0.02	1.59 ± 0.01	1.55 ± 0.03	1.6 ± 0.02

Significantly different from control: * P<0.05; ** P<0.01

Supplementary Table 3 Real-time impedance monitoring following 24 hours treatment with the TJ-disruptor phorbol-12-myristate-13-acetate (PMA) in HepaRG cells: Non-normalized phorbol ester dose-response Table showing effect of PMA on non-normalized data (Z', Rb, z-alpha and Cm impedance parameters), at different time points following PMA challenge. The only statistically significant effect of PMA was on the modeled parameter Rb (cell-cell tight junctions); whilst no significant decrease was measured for either the cell adhesion parameter (z-alpha) or cell membrane capacitance (Cm). See corresponding graphs (**Supplementary Fig. 4**, and **Fig. 4)**.

Supplementary References

1 Anthérieu, S., Chesne, C., Li, R., Guguen-Guillouzo, C. & Guillouzo, A. Optimisation of the HepaRG cell model for drug metabolism and toxicity studies. *Toxicology in Vitro* **26**, 1278-1285 (2012).

2 Giaever, I. & Keese, C. R. A morphological biosensor for mammalian cells. *Nature* **366**, 591-592, doi:10.1038/366591a0 (1993).

3 Wegener, J. & Seebach, J. Experimental tools to monitor the dynamics of endothelial barrier function: a survey of in vitro approaches. *Cell and tissue research* **355**, 485-514, doi:10.1007/s00441-014-1810-3 (2014).

4 Wegener, J., Keese, C. R. & Giaever, I. Electric Cell-Substrate Impedance Sensing (ECIS) as a Noninvasive Means to Monitor the Kinetics of Cell Spreading to Artificial Surfaces. *Experimental Cell Research* **259**, 158-166 (2000).

5 Han, J., Liu, G., Profirovic, J., Niu, J. & Voyno-Yasenetskaya, T. Zyxin is involved in thrombin signaling *via* interaction with PAR-1 receptor. *FASEB* **23**, 4193-4206 (2009).

6 Opp, D. *et al.* Use of electric cell-substrate impedance sensing to assess in vitro cytotoxicity. *Biosensors and Bioelectronics* **24**, 2625-2629 (2009).

7 Keese, C. R., Bhawe, K., Wegener, J. & Giaever, I. Real-Time Impedance Assay to Follow the Invasive Activities of Metastatic Cells in Culture. *BioTechniques* **33**, 842-850 (2002).

8 Atienzar, F. A., Gerets, H., Tilmant, K., Toussaint, G. & Dhalluin, S. Evaluation of impedance-based label-free technology as a tool for pharmacology and toxicology investigations. *Biosensors* **3**, 132-156, doi:10.3390/bios3010132 (2013).

9 Bagnaninchi, P. O. & Drummond, N. Real-time label-free monitoring of adipose-derived stem cell differentiation with electric cell-substrate impedance sensing. *Proc Natl Acad Sci U S A* **108**, 6462-6467, doi:10.1073/pnas.1018260108 (2011).

10 Nipic, D., Pirc, A., Banic, B., Suput, D. & Milisav, I. Preapoptotic cell stress response of primary hepatocytes. *Hepatology* **51**, 2140-2151, doi:10.1002/hep.23598 (2010).

11 Berger et al., Enhancing the functional maturity of induced pluripotent stem cell-derived human hepatocytes by controlled presentation of cell-cell interactions in vitro. Hepatology. 2015 Apr;61(4):1370-81. PMID: 25421237

12 Limonciel et al., Lactate is an ideal non-invasive marker for evaluating temporal alterations in cell stress and toxicity in repeat dose testing regimes. Toxicol In Vitro. 2011 Dec;25(8):1855-62. PMID: 21635945
